# Hydrozoan sperm-specific SPKK motif-containing histone H2B variants stabilise chromatin with limited compaction

**DOI:** 10.1242/dev.201058

**Published:** 2023-01-12

**Authors:** Anna Török, Martin J. G. Browne, Jordina C. Vilar, Indu Patwal, Timothy Q. DuBuc, Febrimarsa, Erwan Atcheson, Uri Frank, Sebastian G. Gornik, Andrew Flaus

**Affiliations:** Centre for Chromosome Biology, School of Biological and Chemical Sciences, University of Galway, Galway H91 TK33, Ireland

**Keywords:** Histone variants, Chromatin, H2B, Sperm-specific histones, SPKK motif

## Abstract

Many animals achieve sperm chromatin compaction and stabilisation by replacing canonical histones with sperm nuclear basic proteins (SNBPs) such as protamines during spermatogenesis. Hydrozoan cnidarians and echinoid sea urchins lack protamines and have evolved a distinctive family of sperm-specific histone H2Bs (spH2Bs) with extended N termini rich in SPK(K/R) motifs. Echinoid sperm packaging is regulated by spH2Bs. Their sperm is negatively buoyant and fertilises on the sea floor. Hydroid cnidarians undertake broadcast spawning but their sperm properties are poorly characterised. We show that *Hydractinia echinata* and *H. symbiolongicarpus* sperm chromatin possesses higher stability than somatic chromatin, with reduced accessibility to transposase Tn5 integration and to endonucleases *in vitro*. In contrast, nuclear dimensions are only moderately reduced in mature *Hydractinia* sperm. Ectopic expression of spH2B in the background of H2B.1 knockdown results in downregulation of global transcription and cell cycle arrest in embryos, without altering their nuclear density. Taken together, SPKK-containing spH2B variants act to stabilise chromatin and silence transcription in *Hydractinia* sperm with only limited chromatin compaction. We suggest that spH2Bs could contribute to sperm buoyancy as a reproductive adaptation.

## Teaser

**Summary:** Hydrozoan spH2B variants containing SPKK motifs package sperm chromatin with limited compaction yet reduce accessibility and downregulate transcription. This could contribute to sperm buoyancy as a reproductive adaptation.

## INTRODUCTION

Male haploid sperm are produced from diploid progenitors during animal spermatogenesis. In this process, progenitor chromatin undergoes major remodelling to produce mature sperm chromatin exhibiting a high degree of condensation and stability compared with somatic chromatin (Ward and Coffey, 1991; Rathke et al., 2014; Hao et al., 2019; Okada, 2022). This is achieved, in part, by sperm nuclear basic proteins (SNBPs) that replace somatic and other variant histones. SNBPs are structurally heterogeneous across species and are classified as histone-type, protamine, protamine-like or SPKK-like motif-containing H2Bs (Török and Gornik, 2018).

**Fig. 1.**
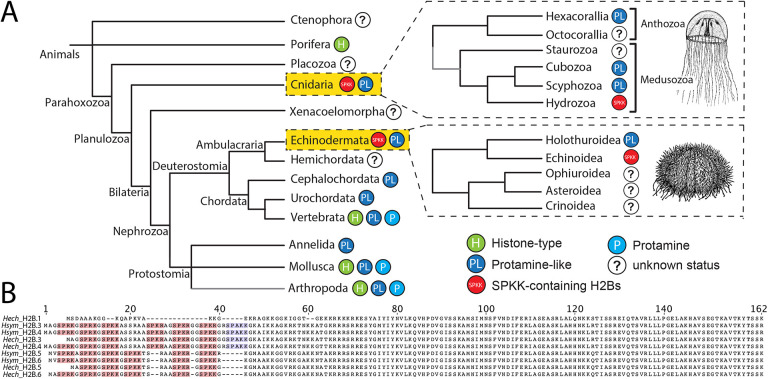
**The evolution of sperm nuclear basic proteins in animals.** (A) Cladogram depicting the distribution of SNBP types in animals as histone-type (H), protamine (P), protamine-like (PL), SPKK-like motif-containing H2Bs (SPKK) and unknown (?). (B) *H. echinata* and *H. symbiolongicarpus* H2B protein sequence alignment highlighting SPKK, SPKR and SPRK (pink), and SPAKK (purple) motifs.

Hydrozoan cnidarians lack genes for protamines. Instead, their genomes encode a number of sperm-specific histone H2B variants (spH2Bs) that are structurally related to a protein family previously characterised in echinoid sea urchins (Busslinger and Barberis, 1985; Poccia and Green, 1992; Rocchini et al., 1996; Ausio et al., 1997; Ausio, 1999; Marzluff et al., 2006; Pérez-Montero et al., 2016; Török et al., 2016) (Fig. 1A). These histones include an extended N-terminal tail with up to seven repeats of a SP(K/R)(K/R) sequence referred to as a SPKK motif that binds with high affinity to DNA minor grooves preferentially in A/T-rich sequences (Churchill and Suzuki, 1989; Suzuki, 1989; Suzuki et al., 1990, 1993). Phosphorylation of the serine on the SPKK motif inhibits its DNA binding (Green and Poccia, 1985; Poccia and Green, 1992).

The SPKK motif-containing dephosphorylated spH2Bs and H1 in the sea urchin *Stronglyocentrotus purpuratus* package uniformly condensed chromatin, resulting in a DNA concentration equivalent to mitotic chromosomes, and increased nucleosome repeat length and inaccessibility to nucleases (Green and Poccia, 1985; Poccia and Green, 1992). This chromatin is rapidly made accessible for transcription by phosphorylation of SPKK motifs after fertilisation (Green et al., 1995).

We have previously shown that spH2Bs are expressed in sperm progenitors in male gonads of the hydrozoan cnidarian *Hydractinia echinata* (Török et al., 2016). Here, we characterise the *Hydractinia* spH2Bs biochemically and functionally using the sibling species *H. symbiolongicarpus*. Our results show that spH2Bs inhibit transcription and stabilise chromatin but with only limited chromatin compaction. We suggest that these characteristics are consistent with a function to facilitate fertilisation in these broadcast-spawning animals.

## RESULTS AND DISCUSSION

### spH2Bs are co-expressed and replace H2B.1 during spermatogenesis but do not increase chromatin compaction

Both *H. echinata and H. symbiolongicarpus* possess several hundred tandemly repeated copies of a single canonical H2B-encoding gene (*H2B.1*) and four sperm-specific H2B variants (*H2B.3*, *H2B.4*, *H2B.5* and *H2B.6*; Fig. 1B). The latter each contain five to seven N-terminal SPKK motifs (Török et al., 2016). The pairs of H2B.3 and H2B.4, and of H2B.5 and H2B.6, are each identical in protein sequence but differ slightly at the nucleotide level and are located at unique chromosomal loci (Török et al., 2016). We refer to all four sperm-specific variants collectively as spH2Bs, except where a specific member is investigated. For *in vitro* experiments, we investigated the properties of *H. echinata* H2B.3 and H2B.6 variants as representatives of spH2B pairs, and compared them with canonical H2B.1.

**Fig. 2.**
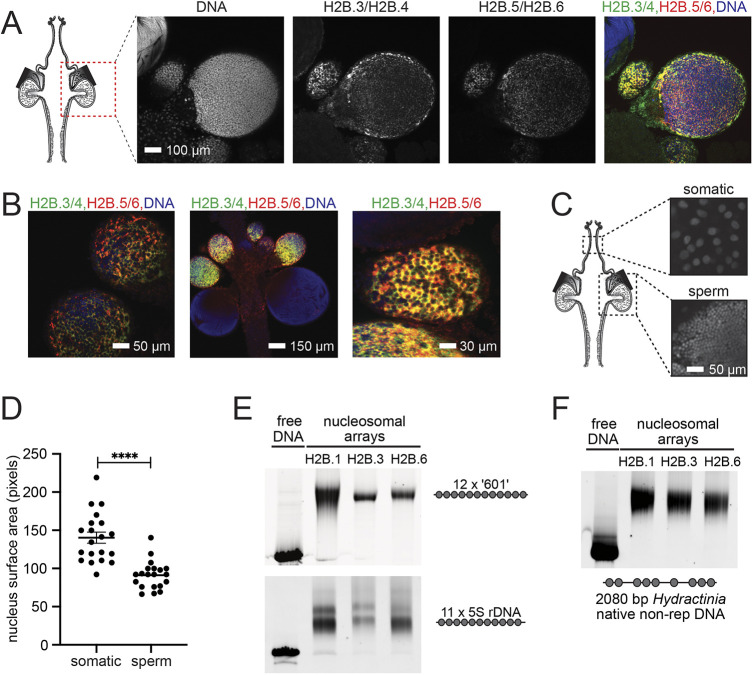
**spH2Bs are co-expressed and replace H2B.1 but do not enhance chromatin compaction.** (A) Sperm progenitor cells with Hoechst-stained DNA, RNA fluorescent *in situ* hybridisation of H2B.3/H2B.4 and H2B.5/H2B.6 expression, and merge. (B) Higher magnification of H2B.3/H2B.4 and H2B.5/H2B.6 RNA fluorescent *in situ* hybridisation showing co-expression of SPKK motif-containing spH2Bs in immature sperm progenitor cells. (C) Hoechst staining of DNA in diploid somatic cells and mature haploid sperm cells for nuclear area measurements. (D) Distribution of nuclear areas for cell types in C (*n*=20 each) showing mean±s.e.m. and ****P<0.0001 (unpaired *t*-test). (E) Native PAGE of nucleosome arrays assembled on 12×177 bp 601 repeat and 11×208 bp 5S rDNA repeat using histone octamers containing H2B.1, H2B.3 or H2B.6. (F) Native PAGE of a 2080 bp non-repetitive *Hydractinia* DNA using the same octamers as in E.

We used fluorescence *in situ* hybridisation to confirm that the expression patterns of spH2Bs in *H. symbiolongicarpus* were equivalent to the previously published *H. echinata* expression. The high nucleotide sequence similarity between *H2B.3* and *H2B.4*, and between *H2B.5* and *H2B.6*, precludes the design of gene-specific cRNA probes (Török et al., 2016) so we treated each pair together in combination. We observed that *H2B.3/4* and *H2B.5/6* were co-expressed in sperm progenitor cells within male gonads of *H. symbiolongicarpus* (Fig. 2A,B) while the expression of *H2B.1* was concomitantly downregulated, consistent with its replacement by spH2Bs during spermatogenesis (Török et al., 2016).

To test whether spH2Bs affect overall genome compaction relative to H2B.1, we measured the nuclear dimensions of sperm cells relative to somatic cells in *H. symbiolongicarpus*. Nuclei of male sexual polyps were stained with Hoechst and confocal images were taken of somatic cells and of cells in the late stage of spermatogenesis (Fig. 2C), then the areas of multiple individual nuclei were calculated. Nuclei of diploid somatic cells had a median area that was 50% larger than haploid mature sperm nuclei, implying only an approximately threefold nuclear volume decrease with the twofold genome size decrease in haploid sperm cells (Fig. 2D). This is consistent with only limited compaction and volume reduction of chromatin involving spH2Bs.

To further investigate the DNA compaction by spH2Bs, we assembled nucleosomal arrays from recombinantly expressed *Hydractinia* histones. Oligonucleosome arrays containing 12 repeats of the well-characterised Widom 601 strong nucleosome positioning sequence (Lowary and Widom, 1998; Dorigo et al., 2003) were assembled with recombinant *Hydractinia* histone octamers containing either H2B.1, H2B.3 or H2B.6. Native PAGE analysis of nucleosomal arrays has been previously used as a sensitive assay of composition and compaction (Bartolomé et al., 1995) but the three nucleosomal arrays did not show any significant difference in the electrophoretic mobility (Fig. 2E). To ensure that this was not an artefact of the 177 bp Widom 601 sequence repeat, we also prepared equivalent oligonucleosome arrays on the 12×208 bp repeat Simpson 5S sequence (Simpson et al., 1985), which had previously been used to demonstrate compaction by SPKK peptides (Khadake and Rao, 1997), and on a native non-repetitive 2080 bp *Hydractinia* genomic AT-rich DNA sequence, with equivalent results (Fig. 2F). We conclude that spH2Bs lead to, at most, only limited chromatin compaction compared with H2B.1 *in vitro*, consistent with our observations *in vivo*.

### Chromatin accessibility is reduced in sperm and *in vitro* assays of spH2B-containing chromatin

**Fig. 3.**
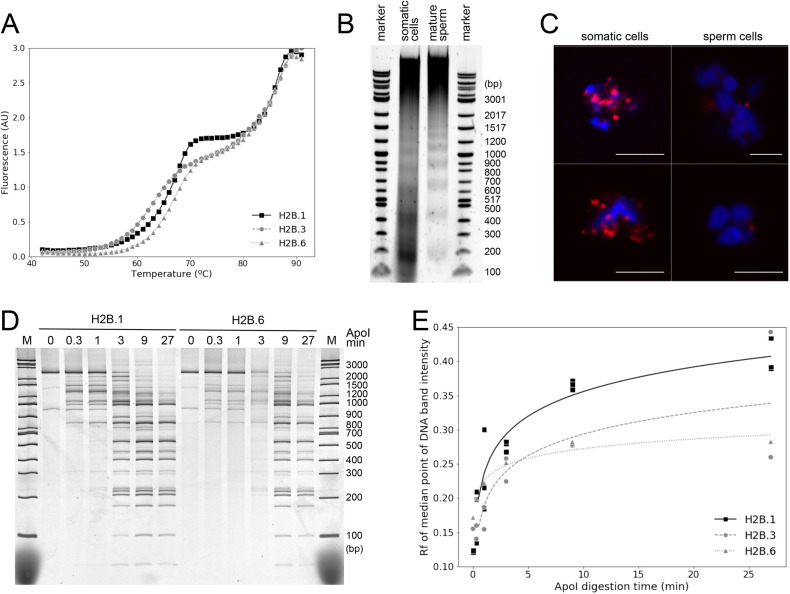
**spH2Bs stabilise chromatin structure and restrict chromatin accessibility *in vivo* and *in vitro*.** (A) Stability of mononucleosomes containing recombinant H2B.1, H2B.3 or H2B.6 measured by temperature dependence of fluorescent dye binding *in vitro*. (B) Difference in nucleosomal repeat for somatic and mature sperm cells after MNase digestion *in vivo*. (C) Relative inaccessibility to Tn5 integrase in ATAC-see (red) of mature sperm cell chromatin compared with somatic cells *in vivo*. Nuclei stained with Hoechst (blue). Tn5-accessible foci in sperm cells are consistent with mitochondria. Representative images from three experiments. Scale bars: 5 µm. (D) Native PAGE of equimolar recombinant H2B.1- or H2B.6-containing nucleosome arrays on 2080 bp *Hydractinia* Piwi promoter DNA digested by ApoI restriction enzyme for indicated times *in vitro*. (E) Quantification of non-denaturing PAGE migration (Rf) for median point of DNA band intensity for ApoI digestion of H2B.1-, H2B.3- or H2B.6-containing nucleosomal arrays from non-denaturing PAGE analyses as in D, showing reduced accessibility of H2B.3 and H2B.6 nucleosomal arrays relative to H2B.1 *in vitro* (H2B.1, *n*=3; H2B.3, *n*=2; H2B.6, *n*=1).

To observe the effect of spH2Bs on nucleosome stability, we assembled mononucleosomes using recombinant *Hydractinia* histone octamers containing H2B.1, H2B.3 or H2B.6 separately and measured their thermal stability (Taguchi et al., 2014). DNA was fully released from all mononucleosomes at 88-90°C, and nucleosomes containing canonical H2B.1 showed a maximum of H2A-H2B dimer dissociation at 70°C (Fig. 3A), consistent with other metazoan canonical H2B-containing nucleosomes (Taguchi et al., 2014). In contrast, nucleosomes incorporating either H2B.3 or H2B.6 showed more progressive thermal dissociation of H2A-H2B dimers reaching a maximum at 78°C.

To probe the impact of spH2Bs on chromatin accessibility *in vivo*, we performed nuclease accessibility assays and observed an increase in nucleosomal repeat lengths from 207±9 bp to 225±5 bp after micrococcal nuclease (MNase) digestion of mature sperm cells compared with somatic cells (Fig. 3B), equivalent to the increase but with a shorter repeat length than sea urchin sperm (Green and Poccia, 1988). Previous investigations also observed reductions in accessibility of linker DNA between spH2B-containing nucleosomes (Green and Poccia, 1988; Hill and Thomas, 1990). We performed ATAC-see on nuclei and observed a strong signal from somatic cells, whereas DNA was inaccessible to Tn5 transposase in mature sperm, except for foci interpreted as mitochondrial DNA that is not packaged by histones (Fig. 3C, Fig. S3). This is consistent with sperm chromatin containing spH2Bs having altered chromatin structure and reduced accessibility *in vivo*.

Finally, we performed parallel *in vitro* ApoI restriction enzyme digestions on nucleosome arrays assembled with H2B.1-, H2B.3- or H2B.6-containing nucleosomes on a native 2080 bp non-repetitive *Hydractinia* DNA fragment. ApoI digestion at eight recognition sites along the DNA was quantitatively slower for H2B.3- or H2B.6-containing arrays than for H2B.1 (Fig. 3D,E), in agreement with the reduction of accessibility observed *in vivo*.

### spH2Bs cause a transcription block and cell cycle arrest in the absence of H2B.1

**Fig. 4.**
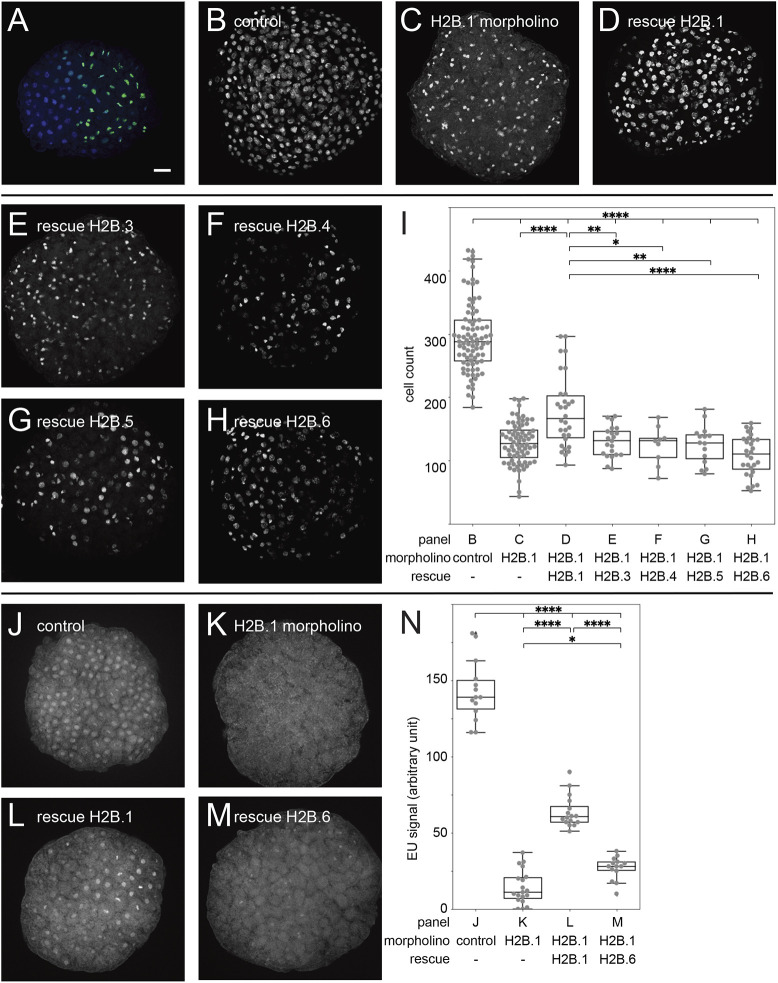
**spH2Bs cause a transcription block and cell cycle arrest in the absence of H2B.1.** (A) GFP expression 7 h after injection of GFP control mRNA into embryos. (B-H) Embryos stained with EdU after injection with (B) control morpholino, (C) H2B.1 translation-blocking morpholino (D), H2B.1-blocking morpholino and morpholino-resistant H2B.1 mRNA, (E) H2B.1-blocking morpholino and H2B.3 mRNA, (F) H2B.1-blocking morpholino and H2B.4 mRNA, (G) H2B.1-blocking morpholino and H2B.5 mRNA, and (H) H2B.1-blocking morpholino and H2B.6 mRNA. (I) Box and whisker plots of EdU-positive cell counts in embryos with *P*-values for one-way ANOVA using 86, 72, 21, 9, 15, 28 and 30 embryos for B-H, respectively. Box plots show first to third quartiles (boxes), minima and maxima within 1.5× interquartile range from respective quartiles (whiskers), and median values (middle bars), along with all sample points (dots). (J) EU incorporation into an embryo injected with control morpholino. (K) EU incorporation into an embryo injected with H2B.1-blocking morpholino. (L) EU incorporation into an embryo injected with H2B.1-blocking morpholino and morpholino-resistant H2B.1 mRNA. (M) EU incorporation into an embryo injected with H2B.1-blocking morpholino and H2B.6 mRNA. (N) Box and whisker plots of EU-positive nuclei per embryo as for I showing *P*-values for one-way ANOVA using 14, 19, 16 and 14 embryos for J-M, respectively (*P<0.1, **P<0.01 and ****P<0.0001). Scale bar: 20 µm.

To test the functional effects of spH2B incorporation in chromatin, we injected *in vitro* transcribed mRNA encoding the full-length sequences of *H2B.3*, *H2B.4*, *H2B.5* and *H2B.6* fused to GFP together into one blastomere of two-cell stage embryos and compared this with injections of mRNA encoding GFP alone as a control. All embryos showed GFP fluorescence 6 h after injection in 50% of cells (Fig. 4A and Fig. S2A). Animals injected with combined spH2B-GFP fusion mRNAs exhibited nuclear fluorescence, whereas control *GFP* alone was cytoplasmic. Embryos developed into planula larvae within 2-3 days, similar to untreated *Hydractinia* embryos (Fig. S2B).

As there are some 700 copies of the canonical *H2B.1* gene in the *Hydractinia* haploid genome but only single copies of each spH2B variant (Török et al., 2016), we hypothesised that the injected mRNAs encoding spH2Bs would be outnumbered by endogenous *H2B.1* transcripts. Therefore, we co-injected a *H2B.1*-specific translation blocking morpholino together with the mRNAs for the four spH2B variants. We used EdU incorporation to monitor the number of replicating nuclei per embryo by confocal microscopy. Control morpholinos had no effect on the embryos (Fig. 4B), and injecting the morpholino alone led to cell cycle arrest and death of all embryos within a few cell cycles (Fig. 4C). This could be partially rescued by co-injection of morpholino-resistant *H2B.1* encoding mRNA (Fig. 4D), confirming the specificity of the morpholino (Fig. S1B).

We then injected *H2B.3*, *H2B.4*, *H2B.5* and *H2B.6* mRNAs individually in combination with the *H2B.1*-specific morpholino. This also caused cell cycle arrest equivalent to the injection of the morpholino alone, showing that spH2Bs cannot substitute for H2B.1 (Fig. 4E-I). Finally, we used ethynyl uridine (EU) incorporation to quantitate nascent RNA levels and found them to be significantly lower in *H2B.6* mRNA-injected embryos than in embryos co-injected with the *H2B.1* rescue mRNA in morphants (Fig. 4J-N). Together, the mRNA injection experiments are consistent with spH2B incorporation in chromatin in place of H2B.1 leading to a cell cycle arrest by blocking replication and transcription.

In summary, *in vivo* and *in vitro* assays showed that sperm-specific histones H2B.3, H2B.4, H2B.5 and H2B.6 containing N-terminal SPKK motifs increase chromatin stability and reduce DNA accessibility compared with chromatin assembled with canonical H2B.1. The spH2B histones cannot rescue knockdown of H2B.1. Taken together, our observations are consistent with silencing of replication and transcription resulting from networks of linker DNA binding of spH2Bs in sperm, in line with earlier investigations with structurally similar H2B variants in sea urchin sperm. Intriguingly, spH2Bs are only known in distantly related cnidarians and echinoderms, so spH2Bs may have arisen convergently in these taxa given the suggested ease of evolving SPKK motifs (Malik et al., 2002; Török and Gornik, 2018).

Protamine-type SNBPs generate nuclei that are up to 40-fold more compact than somatic histone-packaged chromatin (Ward and Coffey, 1991; Okada, 2022), which has been suggested to improve sperm motility (Levitan and Petersen, 1995; Champroux et al., 2016). Sea urchin sperm nuclei using SPKK motif-containing spH2Bs and H1 have uniform compaction equivalent to metaphase chromosomes (Green and Poccia, 1985). Our observations using both *in vivo* and *in vitro* assays show *Hydractinia* spH2Bs share many features of sea urchin spH2Bs but more limited chromatin compaction. The ∼250 bp nucleosomal repeat of sea urchin sperm is one of the longest recorded (Poccia and Green, 1992; Widom, 1992), whereas we measure a more moderate repeat length in *Hydractinia* sperm.

*Hydractinia* colonies are dioecious and grow individually on the surface of hermit crab shells. Their spawning is light induced but the distance to a potential mate depends on the behaviour of host hermit crabs. Eggs are near neutrally buoyant upon spawning, sinking only very slowly to the bottom in calm water (Movie 1) and remaining suspended under turbulent conditions. This feature probably prevents egg loss in the sediment, but fertilisation becomes unlikely unless sperm have a similar buoyancy. In contrast, echinoids such as sea urchins undertake fertilisation on the sea floor.

Conversely, after fertilisation, *Hydractinia* zygotes become strongly negatively buoyant (Movie 2), potentially to protect the embryo from predation in the water column and allow the larvae to attach to benthic hermit crab shells. As chromatin contributes a major part of total sperm biomolecule composition, we speculate that spH2Bs could contribute to *Hydractinia* sperm buoyancy by providing reduced accessibility without increasing compaction. Less dense sperm would increase fertilisation likelihood and could be the selective pressure that drove the evolution of hydrozoan chromatin mediated by spH2Bs.

## MATERIALS AND METHODS

### Animal culture

*Hydractinia symbiolongicarpus* colonies were cultured in artificial seawater at 18°C under 14h:10 h light-dark regimes and fed *Artemia franciscana* nauplii four times a week, and ground oyster once per week. Spawning is light induced, occurring synchronously in both sexes (Frank et al., 2020). Fertilised eggs were collected for microinjection and developed at room temperature into planula larvae within 3 days. Male polyps were harvested from mature colonies.

### Microinjection

Injection needles were prepared from glass capillaries (Narishige GD-1 1×90 mm) using a microneedle puller (Narishige PN-31) with settings of heat 560, pull 70, velocity 75 and time 150. Microinjection was carried out immediately after fertilisation in one-cell stage embryos. Equal volumes of 500 ng/μl morpholino and 0.5 μg/μl mRNA in ∼5% of the zygote volume were injected into embryos as indicated. Embryos were placed in a small Petri dish lid with a 200 μm plankton net attached to avoid any movement. Injections were completed before the first cell division.

### Genomic DNA extractions

Genomic DNA was extracted from polyps by separating them from adult colonies using surgical scissors and repeatedly washing them in sterile filtered artificial seawater. The animal tissue was then disrupted in 1 ml of 100 mM Tris HCl (pH 8), 1% SDS and 50 mM EDTA lysis buffer using a plastic pestle. 2 µl of 10 mg ml^−1^ RNaseA (ThermoFisher EN0531) and 2 µl RNaseT1 1000 U ul^−1^ (ThermoFisher EN0541) were added and incubated at 37°C for 1 h. 2 µl of Proteinase K (25 mg ml^−1^, Qiagen, 19133) was added and the solution was incubated at 50°C for 2 h. Equal volumes of phenol (pH 8) were added and the aqueous phase containing DNA was separated by centrifugation. This was repeated with phenol again and then with chloroform. The genomic DNA was precipitated with 0.1 volumes of 5 M NaCl and 2.5 volumes of absolute ethanol, then washed with 70% ethanol three times. The pellet was air-dried at room temperature and resuspended in 10 mM Tris (pH 8.0) and 1 mM EDTA.

### Micrococcal nuclease (MNase) assay

Polyps were cut from adult colonies using surgical scissors. Sperm were extracted from ∼60 mature gonads using a 23^5^/_8_″ gauge syringe needle into 20 µl of 4% MgCl_2_·6H_2_O. 1 ml of 10 mM DTT with complete protease inhibitor (Roche 11697498001) was added and the samples were incubated on ice for 30 min. Nuclei were centrifuged at 16,000 ***g*** at 4°C for 25 min and the pellet was resuspended in 800 µl of 20 mM Tris (pH 7.5), 15 mM NaCl, 60 mM KCl, 1 mM CaCl_2_, 5 mM MgCl_2_, 300 mM sucrose and 0.4% NP40 containing 0.0125 units of RNAse T1 (ThermoFisher EN0541). Aliquots of 200 µl were warmed to 37°C for 1 min then 0, 0.02, 0.2 and 0.4 units of MNase (NEB M0247S) were added, mixed and incubated at 37°C for 3 min. The reaction was stopped with 6.8 µl 100 mM EDTA and 4% SDS. Then 5 µl of Proteinase K (25 mg ml^−1^, Qiagen 19133) was added and incubated at 55°C for 1 h. DNA was phenol-chloroform extracted then resuspended in 15 µl H_2_O and run on a 2% agarose gel containing SYBRSafe DNA stain (Invitrogen, S33102) at 100 V for 40 min and visualised using a UV box.

Gels were analysed using ImageJ 1.53k to define a standard curve for mean migration of flanking ladder bands, then MNase peak fragment sizes were interpolated. Nucleosomal repeat lengths were estimated from MNase product fragments 2-7 and are expressed as mean±s.d.

### Fluorescent *in situ* hybridisation

Animals were anaesthetised by incubation in 4% MgCl_2_ in 50% artificial sea water for 30 min then polyps were excised and soaked in the same solution for 10 min in methanol to stop shrinkage then fixed in 4% paraformaldehyde in 100 mM HEPES (pH 7.5), 4 mM MgSO_4_ and 140 mM NaCl overnight at 4°C. Polyps were washed three times in 0.1% Tween 20 in 10 mM Na_2_HPO_4_, 1.8 mM KH_2_PO_4_, 136 mM NaCl and 2.7 mM KCl (pH 7.5) (PBST) then dehydrated stepwise in 25%, 50% and 75% methanol for 5 min each and finally in 100% methanol for 15 min before storage at −20°C or immediate use. Polyps were rehydrated by washing in reverse steps of methanol followed by three washes in PBST for 5 min. Polyps were then fixed stepwise in triethylamine (TEA), 0.06% acetic acid in TEA, 0.12% acetic acid in TEA and PBST for 5 min each. Finally, polyps were incubated in 4% paraformaldehyde in PBS for 20 min followed by three washes in 0.3% Triton X-100 in PBS (PBSTx) for 5 min each.

For blocking, the polyps were placed in 500 μl 2 mg ml^−l^ yeast tRNA in PBSTx for 10 min. An equal volume of hybridisation buffer comprising 50% formamide, 5×SSC, 0.1 mg ml^−1^ heparin, 0.1 mg ml^−1^ yeast tRNA, 0.1% Tween20 was added to the blocking solution for 10 min. The solution was then replaced with hybridisation buffer and pre-hybridisation was carried out overnight at 50-52°C.

Digoxigenin (DIG) and fluorescein (FITC) labelled probes at 40 ng ml^−1^ were added to the hybridisation buffer and incubated overnight at 50-52°C on a shaker. Polyps were then incubated in 2% H_2_O_2_ for 60 min and washed in PBSTx for 10 min. Blocking was carried out using 0.1 M Tris-HCl (pH. 7.5), 0.15 M NaCl and 0.5% blocking reagent.

For detection, anti-DIG conjugated to horseradish peroxidase antibody (Roche 11207733910) was diluted at 1:1000 in blocking buffer and polyps were incubated in overnight at 4°C then washed three times in PBSTx for 5, 30 and 10 min sequentially. The DIG-labelled probe was detected using a Tyramide Signal Amplification kit (PerkinElmer NEL744001KT). 1 μl of Cyanine 3 Amplification Reagent was diluted in 50 μl of 1× Plus Amplification diluent by placing the solution on the polyps for 20-30 min then washed three times in PBSTx for 10 min and incubated in 2% H_2_O_2_ for 60 min before washing in PBSTx for 10 min. The same steps were used to detect the FITC-labelled probe by diluting 1 μl of Fluorescein Amplification Reagent in 50 μl of 1X Plus Amplification diluent (PerkinElmer NEL741001KT). For DNA detection, nuclei were stained in 10 ng/μl Hoechst 33258 (Sigma B2883) in PBSTx for 15 min.

The animals were then mounted on microscopic glass slides using Fluoroshield mounting medium (Sigma F4680), sealed with nail polish and stored at −20°C. Fluorescent *in situ* hybridisation images were taken on Olympus FV1000 inverted confocal microscope.

### Capped and polyadenylated mRNA synthesis

For mRNA synthesis, the desired fragment including 100 bp 5′-UTR and the coding sequence of the histone gene was amplified by PCR from genomic DNA. Linker sequences and GFP tags were added to the construct by Gibson assembly. The assembled fragment was re-amplified by PCR, with primers containing T7 promoter necessary for RNA synthesis. HiScribe T7 ARCA mRNA kit (NEB E2060S) was used for mRNA synthesis with 10 μl of 2× ARCA/NTP mix, 1 μg of template DNA, 2 μl of T7 RNA Polymerase mix and nuclease-free water to a final volume of 20 μl. Reactions were gently mixed and incubated at 37°C for 1-2 h. 2 μl DNaseI was added to the mixtures and incubated for a further 15 min. PolyA tailing was carried out in a total volume of 100 μl with 20 μl of *in vitro* transcription reaction, 65 μl of nuclease-free water, 10 μl of 10x PolyA Polymerase Reaction Buffer and 5 μl of PolyA polymerase. Reactions were mixed and incubated at 37°C for 30 min. In order to purify mRNA, a 0.5 volume of 7.5 M LiCl and 10 mM EDTA was added to each sample and incubated at −20°C for 30 min. Tubes were centrifuged at 4°C for 15 min at full speed. Supernatant was removed and pellets were rinsed with 500 μl ice-cold ethanol. Tubes were centrifuged again at 4°C for 10 min at full speed. Ethanol and residual liquid was carefully removed using a sharp tip then heated to 65°C for 5-10 min to completely dissolve RNA. After this, mRNA concentrations were measured and the tubes were stored at −80°C. mRNA could be visualised and analysed on formaldehyde denaturing gels.

### EdU staining of S-phase cells

EdU visualisation was performed using a Click-iT EdU Alexa Fluor 488 Imaging kit (ThermoFisher C10337). The solutions were prepared according to the manufacturer's instructions. Fluorescence excitation and emission maxima for Alexa Fluor 488 were 495 and 519 nm, respectively. For EdU staining, animals were incubated in EdU solution for 30 min at a concentration of 150 μM. After incubation, fluorescent *in situ* hybridisation was performed as described above when it was necessary. Animals were then fixed in 4% PFA in 100 mM HEPES (pH 7.5), 4 mM MgSO_4_ and 140 mM NaCl overnight at 4°C. After this, animals were washed twice in 3% BSA/PBSTx (0.1%) for 15 min each. The sample was permeabilised by incubation in 0.5% PBSTx for 1 h. Animals were then washed twice for 10 min in 3% BSA/PBSTx (0.1%). Staining cocktail containing 430 μl of 1× reaction buffer, 20 μl CuSO_4_, 1.2 μl Alexa Fluor 488 azide and 50 μl of reaction buffer additive in a total volume of 0.5 ml was prepared. The reaction cocktail was then added to the animals to cover them entirely and they were then incubated in the dark for 30 min at room temperature then washed four times in 3% BSA/PBS Triton (0.1%) for 15 min each. Nuclei were stained in 10 ng μl^−1^ Hoechst 33258 (Sigma B2883) in PBSTx for 15 min followed by one wash in PBSTx for 10 min. Samples were mounted on microscopic glass slides using Fluoroshield mounting medium (Sigma F4680), sealed with nail polish and stored at −20°C. Images were taken on Olympus FV1000 inverted confocal microscope.

### EU staining to detect nascent RNA

EU visualisation was performed using a Click-iT RNA Imaging Kit (ThermoFisher C10086). The solutions were prepared according to the manufacturer's instructions. For EU staining embryos were incubated in EU solution for 1 h at a final concentration of 0.5 mM and fixed in 4% PFA in HEPES for 1 h at room temperature. Embryos were then washed in PBS for 5 min followed by quick washes in increasing concentrations of methanol. Animals were first placed in 25% methanol v/v in PBS followed sequentially by 50%, 75% and 100% methanol. The embryos were then rehydrated by washing in decreasing concentrations of methanol. First in 75% methanol, then in 50% methanol and finally in 25% methanol for a few seconds each. Embryos were washed once again in PBS for 5 min. To permeabilise the samples, the embryos were incubated in 0.5% TritonX-100 in PBS for 15 min at room temperature. The permeabilisation buffer was removed and the embryos were washed once with PBS for 5 min. Staining cocktail was prepared to a total volume of 250 μl containing 214 μl of 1× Click-iT RNA reaction buffer, 10 μl CuSO_4_, 1 μl Alexa Fluor 488 azide and 25 μl Click-iT reaction buffer additive. The reaction cocktail was added to the embryos to cover them entirely. The animals were incubated for 30 min at room temperature, protected from light. After incubation, animals were washed four times in PBS for 15 min each. Nuclei were stained in 10 mg ml^−1^ DAPI in PBS for 15 min. Animals were washed in 0.3% TritonX-100 in PBS for 10 min. Samples were then mounted on microscopic glass slides using Fluoroshield mounting medium (Sigma F4680), sealed with nail polish and stored at −20°C. Images were taken on Olympus FV1000 inverted confocal microscope.

### Microscopy

An Olympus SZX7 stereomicroscope was used for animal observation, colony maintenance and injection. Fluorescence microscopy was carried out using an Olympus BX51 compound microscope. Images were processed with the Olympus CellD software package. Confocal microscopy was carried out using Olympus Fluoview 1000 software with an inverted IX71 microscope. In most cases 20× and 40× objectives, and Alexa Fluor 405, Alexa Fluor 488 or Alexa Fluor 594 were selected. The zoom ratio was then set to 1× with the appropriate channels (DAPI, FITC and Cy3) and the highest scan speed. PMT voltage was increased to improve the sensitivity, and the gain and offset values were also optimised independently when it was necessary to brighten or darken the image. The XY observation mode was used to perform repeated scanning. After an image was displayed, the area of interest was selected and scan speed was lowered. In order to reduce noise, the Kalman accumulation function was applied. Images were then acquired and saved. In order to observe the desired multiple sections the *z*-position was also adjusted. The stacks of sections could then be projected together. Image analysis was carried out using ImageJ Software.

### Cell counting and image analysis

The Automated Counting feature of ImageJ/Fiji (https://fiji.sc/) was used to count the number EdU-positive nuclei in confocal *z*-stack projections as a proxy for numbers of cells per embryo with two or three independent technical replicates each. To count EU-positive nuclei, confocal *z*-stack projections were used equivalently. Briefly, coloured *z*-stack images (RGB) were first converted into greyscale before proceeding through the following settings: ‘Edit – Options – Conversations’ to ‘scale when converting’. Images were then converted to greyscale using ‘Image - Type - 16-bit’. All structures to be counted were highlighted according to the following settings: ‘Image - Adjust – Threshold’. The sliders were then used to highlight structures, and settings were applied to create a binary version of the image with pixel intensities black=0 and white=255. When particles overlapped, the ‘Process - Binary – Watershed’ function was applied to separate then by adding a one pixel thick line. After this, the ‘Analyze - Analyze Particles’ function was used. In order to avoid counting noise, particle size was adjusted excluding noise-derived particles. The outlines of cell nuclei were then displayed together with a summary table showing the number of cells counted. These results were then used for statistical analysis. Data were normalised and *P* values were calculated using one way ANOVA tests in the SPSS software package (*P<0.1, **P<0.01, ***P<0.001 and ****P<0.0001).

### Analysis of nuclear areas

Nuclei of intact male sexual polyps were stained by Hoechst and confocal images from early to late stages of spermatogenesis were taken, along with epithelial somatic cells from the surface of the body column with the same magnifications and settings. The perimeter of 20 nuclei in an optical section of each sample was defined manually and the pixels counted as a measurement of area. The *P* value was calculated using a two-tailed *t*-test in the SPSS software package with ****P<0.0001.

### Morpholino mediated knockdown

Morpholino oligos were designed around the start codon of the gene of interest using Gene Tools software (http://www.gene-tools.com) (Fig. S1A). Morpholino oligos were diluted to the recommended concentrations and injected into one-cell stage embryos: control morpholino sequence, CCTCTTACCTCAGTTACAATTTATA; H2B.1 morpholino sequence: GCTGCTGCGTCAGACATGGTTAAAT.

### Assay of transposase-accessible chromatin with visualisation (ATAC-see)

Sperm were obtained as described above. Somatic cells were harvested from pronase-dissociated feeding polyps that do not contain germ cells. A hyperactive Tn5 transposase was loaded with DNA adaptors that selectively bind to only accessible chromatin (Buenrostro et al., 2013). Tn5 transposase assembly was carried out by resuspending Tn5ME-AATTO590, Tn5ME-B-ATTO590 and Tn5MErev oligos in nuclease-free water to a final concentration of 100 μM. Equimolar amounts of Tn5MErev/Tn5ME-A-ATTO590 and Tn5MErev/Tn5ME-B-ATTO590 were mixed in separate 200 μl PCR tubes and incubated at 95°C for 5 min then cooled down slowly by turning off the thermocycler. The assembly of transposase solution was carried out in dark using 0.25 volumes of Tn5MErev/Tn5ME-A-ATTO590+Tn5MErev/Tn5ME-B-ATTO590 at 50 μM final concentration, 0.4 volume of sterile 100% glycerol, 0.12 volume of sterile filtered 2× dialysis buffer [100 mM HEPES–KOH (pH 7.2), 0.2 M NaCl, 0.2 mM EDTA, 2 mM DTT, 0.2% Triton X100 and 20% glycerol), 0.1 volume of 50 μM SL–Tn5 and 0.13 volumes of water. Reagents were gently mixed and incubated at room temperature for 1 h, allowing the oligos to anneal to Tn5. The transposase solution was stored at −20°C. Slide preparation and fixation was carried out by fixing *Hydractinia* sperm cells and pronase-dissociated somatic cells with 1% formaldehyde for 10 min at room temperature. Sperm cells were fixed on glass coverslips using Cytospin, but this could not be performed with somatic cells as it damaged their structure. ATAC-see staining was carried out by permeabilising with lysis buffer [10 mM TrisCl (pH 7.4), 10 mM NaCl, 3 mM MgCl_2_ and 0.01% Igepal CA-630] for 10 min at room temperature. The samples were rinsed with 1×PBS twice and placed in a humid chamber box at 37°C. The transposase mixture solution comprising 25 μl 2× TD buffer with final concentration of 100 nM Tn5ATTO-59ON in a volume of 50 μl was placed on the cells and incubated for 30 min at 37°C. The slides were washed with 1×PBS containing 0.01% SDS and 50 mM EDTA for 15 min three times at 55°C. Nuclei were stained in 10 ng μl^−1^ Hoechst 33258 (Sigma B2883) then mounted using Fluoroshield medium (Sigma F4680) for confocal imaging. Twenty representative images each for feeding polyp and sperm cells were captured, and signal was quantitated after maxentropy thresholding for 405 nm and 618 nm excitation images for Hoeschst and ATAC-see, respectively, using ImageJ 1.53v.

### Recombinant histone expression

All histone genes except H4 were amplified from *Hydractinia* genomic DNA using degenerate primers and subcloned into pET3a. The H4-coding sequence was optimised and synthesised in pD451 (Table S1). *E. coli* Rosetta2 pLysS or Star pRIL cells chosen for optimal expression were transformed with expression plasmids and plated on LB agar containing appropriate antibiotics then grown overnight at 37°C. Colonies were transferred to 1 l of 2YT media supplemented with antibiotics and grown until OD600 0.6-0.8. Expression was induced by the addition of IPTG to a final concentration of 0.4 mM. Cultures were grown for a further 4 h at 37°C and shaking at 180 rpm. Cells were then harvested by centrifugation at 5000 ***g*** for 15 min and cell pellets were stored at −20°C.

### Recombinant histone purification

The cell pellet containing histone inclusion bodies were allowed to thaw and was resuspended in 30 ml histone wash buffer [50 mM Tris-HCl (pH 7.5), 100 mM NaCl, 5 mM β-mercaptoethanol and 1 mM benzamidine hydrochloride]. Cell suspensions were sonicated in a Branson Sonifier 250 with 12 mm tip on ice for 1 min at 40% amplitude with 5 s on pulses and 10 s off pulses, and the lysate was centrifuged at 30,000 ***g*** for 15 min. The resulting pellet was washed several times with histone wash buffer, resuspended in 0.5 ml DMSO and incubated at room temperature for 30 min. The inclusion bodies were further solubilised by the addition of 5 ml of unfolding buffer [20 mM Tris-HCl (pH 7.5), 7 M Guanidine HCl and 10 mM DTT] and incubated on rollers for 1 h at room temperature. Following this, histone purification buffer A [50 mM Tris-HCl (pH 7.5), 7 M urea and 1 mM EDTA) was added to a final volume of 20 ml and centrifuged at 35,000 ***g*** for 20 min. The resulting supernatant was centrifuged for 20 min at 35,000 ***g***. This final supernatant was then added to 55 ml of histone purification buffer A. The histone solution was passed through a PVDF filter with 0.2 μm pore size attached to a vacuum bottle. Samples were then loaded onto a 5 ml SP Sepharose HP column using a FPLC system. Histones were eluted with a linear gradient of increasing histone purification buffer B [50 mM Tris-HCl (pH 7.5), 7 M urea, 1 mM EDTA and 2 M NaCl].

### SDS polyacrylamide gel electrophoresis

SDS polyacrylamide gels were made to a final acrylamide concentration of 15% acrylamide with 1:37.5 bis-acrylamide. Samples were resuspended in the presence of 1× protein gel loading buffer and heated to 95°C for 5 min before loading. Gel electrophoresis was carried out by applying a constant 100 V in 1× TG buffer (30 g l^−1^ Tris and 144 g l^−1^ glycine). Protein bands were visualised by Coomassie Blue staining.

### Histone octamer refolding by salt dialysis

Equal quantities of lyophilised histones were resuspended in unfolding buffer to a final concentration of 2 mg ml^−1^. Denaturing resuspension was carried out at room temperature on a roller for 1 h then centrifugation at 18,000 ***g*** for 10 min. The concentration of each histone supernatant was determined using calculated extinction coefficients (Expasy ProtParam; https://web.expasy.org/protparam/). The four histones were mixed in equimolar ratios and the volume adjusted to a final protein concentration of 1 mg ml^−1^. This mixture was placed in an 8000 MWCO dialysis bag and twice dialysed against 600 ml of refolding buffer [10 mM Tris-HCl (pH 7.5), 2 M NaCl, 1 mM EDTA and 5 mM β-mercaptoethanol] at 4°C for 3 h each followed by a third overnight dialysis. After concentration to 1 ml using a Millipore Ultrafree 10 K MWCO concentrator, histone octamers were purified by size exclusion chromatography using a Superdex 200 column equilibrated in refolding buffer. Fractions containing all four histones at equimolar ratios were confirmed by SDS polyacrylamide gel electrophoresis (SDS-PAGE) and pooled then concentrated to ∼10 μM using a Millipore Ultrafree 10K MWCO concentrator.

### DNA preparation by plasmid digestion

Plasmids containing 12×177 bp 601 (Dorigo et al., 2003) and pCL3 containing 11×208 bp 5S rDNA DNA repeats (Logie and Peterson, 1997) were transformed into *E. coli* TOP10 cells then grown in large scale LB media cultures with appropriate antibiotics. Plasmids were extracted from harvested cells by using ThermoFisher GeneJet plasmid DNA purification kits (ThermoFisher K0481).

The 601 repeat insert was isolated by digesting purified plasmid at 37°C overnight using 0.34 NEB units μg^−1^ of EcoRV. The 5S repeat insert was isolated by digesting pCL3 with EcoRV, BglI and BstXI at 37°C overnight using 0.34 NEB units μg^−1^ of each enzyme. The 2.1 kb and 2.3 kb inserts were isolated from smaller plasmid fragments after electrophoresis in 0.7% agarose gels run in 1× TAE buffer [40 mM Tris-acetate (pH 8.0) and 1 mM EDTA) using a ThermoFisher GeneJet gel extraction kit (ThermoFisher K0692).

Finally, DNA fragments were loaded onto a 5 ml MonoQ ion exchange column (Cytiva 17516601) and eluted by a linear gradient of 20 mM Tris-HCl (pH 7.5), 0.1 mM EDTA and 2 M NaCl over five column volumes. The desired peak fractions were identified by 1% agarose gel electrophoresis, then ethanol precipitated and the pellet was resuspended in 10 mM Tris-HCl (pH 7.5).

### DNA preparation by PCR

Two 96-well plates of identical PCR reactions with 400 pg µl^−1^ template plasmid and 400 µM dNTPs in Taq buffer [10 mM Tris-HCl (pH 9.0), 50 mM KCl, 1.5 mM MgCl_2_ and 0.1% Triton X-100) with were amplified using Taq polymerase with 800 nM primers. Template plasmids containing a 2080 bp non-repetitive region of the *H. echinata* Piwi1 promoter (Table S1; F, CAGATGATCCGCAGACAATAG; R, AAATGTAATGAAAATTTTCGTAATTA) or the Widom ‘601’ sequence (F, CTGCAGAAGCTTGGTCCC; R, ACAGGATGTATATATCTG) were used for the 2080 bp and 147 bp fragments, respectively. PCR wells were pooled and ethanol precipitated by adding 0.1 volumes of 3 M sodium acetate (pH 5.2) and 2.5 volumes of absolute ethanol then centrifuging at 11,000 ***g*** at 4°C for 30 min. The DNA pellet was resuspended in 2 ml of DNA-binding buffer A [20 mM Tris-HCl (pH 7.5) and 0.1 mM EDTA). Finally, DNA was purified by ion exchange chromatography as described above.

### Nucleosome assembly by salt dialysis

To assemble 250 pmol of nucleosomes in a volume of 80 μl, purified octamer was mixed with the appropriate DNA in an equimolar ratio of nucleosome sites in a solution containing a final concentration of 20 mM Tris-HCl (pH 7.5) and 2 M NaCl. The reaction mixture was transferred into a custom-made miniature dialysis block pre-equilibrated at 4°C with 8000 MWCO dialysis membranes. Samples were dialysed against solutions containing 10 mM Tris-HCl (pH 7.5) and 1 mM EDTA with consecutively 1.4 M, 1.2 M, 0.8 M and 0.6 M NaCl for at least 2 h each, and then finally against 10 mM Tris-HCl (pH 7.5) overnight. Nucleosomal arrays were visualised by mixed agarose-polyacrylamide native gel electrophoresis; mononucleosomes were visualised by native PAGE.

### Thermal stability assay by fluorescent dye binding

A 20 µl reaction mix was prepared by mixing 36 pmol of nucleosomes assembled on 147 bp Widom 601 DNA with freshly diluted 5× SyPro Orange with 0.1 mM NaCl, 20 mM Tris-Cl (pH 7.5) in a 96-well MicroAmp Fast optical plate. Thermal unfolding analysed on StepOne Plus Thermal Cycler as described previously (Taguchi et al., 2014).

### Mixed agarose-polyacrylamide native gel electrophoresis

Gels comprising 1% agarose and 2% acrylamide with 1:37.5 bisacrylamide in 0.2× TBE were heated to dissolve agarose, where 1× TBE is 89 mM Tris (pH 7.5), 89 mM borate and 2 mM EDTA. The solution was allowed to cool to ∼40°C before adding 0.1% TEMED and ammonium persulphate to 0.1% then pouring into pre-warmed 16 cm×20 cm glass plates with 1 mm spacers. Polymerised gels were pre-equilibrated for 145 min at 250 V in 0.2× TBE Buffer. Samples were mixed with 0.7 volumes of 10 mM Tris-HCl (pH 7.5) and 6% sucrose. Electrophoresis was performed under the same conditions as pre-equilibration.

### Nucleosome array digestion by ApoI

Nucleosome arrays were digested with ApoI restriction enzyme by mixing 15 pmol assembled nucleosomal array in 50 µl volume with 1× NEB CutSmart buffer and 7.5 units of restriction enzyme at 37°C. 10 µl samples were withdrawn at each time point and mixed rapidly with 33% phenol and 33% chloroform to stop the reaction. The aqueous phase was separated by centrifugation then ethanol precipitated, and the DNA pellet was resuspended in 1× DNA loading dye. Digestion was visualised by non-denaturing PAGE.

### Native/non-denaturing PAGE of nucleosomes and DNA

A 6% acrylamide with 1:37.5 bisacrylamide native polyacrylamide gel was cast and left for 1 h at room temperature for complete polymerisation. The gel was thermally equilibrated for 1 h at 4°C before being pre-run for 3 h at 250 V and 4°C in 0.2× TBE. 4 pmol of nucleosome or DNA in 5% sucrose was loaded per lane and the gel was run for a further 3 h at 250 V at 4°C in 0.2× TBE.

### Imaging of native/non-denaturing polyacrylamide gels

Gels with unlabelled DNA were stained with 10 ng ml^−1^ ethidium bromide for 15 min then scanned using a Fuji FLA5100 or BioRad Pharos FX fluorescent imager with the appropriate laser and filter settings for Cy3, Cy5 or ethidium bromide.

### Analysis of non-denaturing PAGE migration of DNA

Individual lanes from immediately below the well to the smallest DNA band were cropped from TIFF images, read pixelwise and summed by row to obtain the signal at each point down the lane. Background was estimated from the region of 97% migration and subtracted, then the cumulative intensity of signal at each point was calculated from the bottom. This was normalised to the maximum signal for the lane to avoid effects from different loadings in lanes. The relative migration of the 50th percentile for the normalised cumulative signal was extracted as the Rf of median DNA band intensity and plotted versus ApoI digestion time. Time series data for histone octamers containing each histone variant were fitted to a log normal function using curve_fit from the optimise module of SciPy (https://scipy.org). All data were plotted using Seaborn (https://seaborn.pydata.org) with appropriate matplotlib parameters.

## Supplementary Material

10.1242/develop.201058_sup1 Supplementary information
