# Supplementary material for: Hydrozoan sperm-specific SPKK motif-containing histone H2B variants stabilise chromatin with limited compaction
Source: Development. 2023 Jan 12;150(1):dev201058. doi: 10.1242/dev.201058 (PMC9903204; doi:10.1242/dev.201058)
Supplement: Supplementary information [file develop-150-201058-s1.pdf]

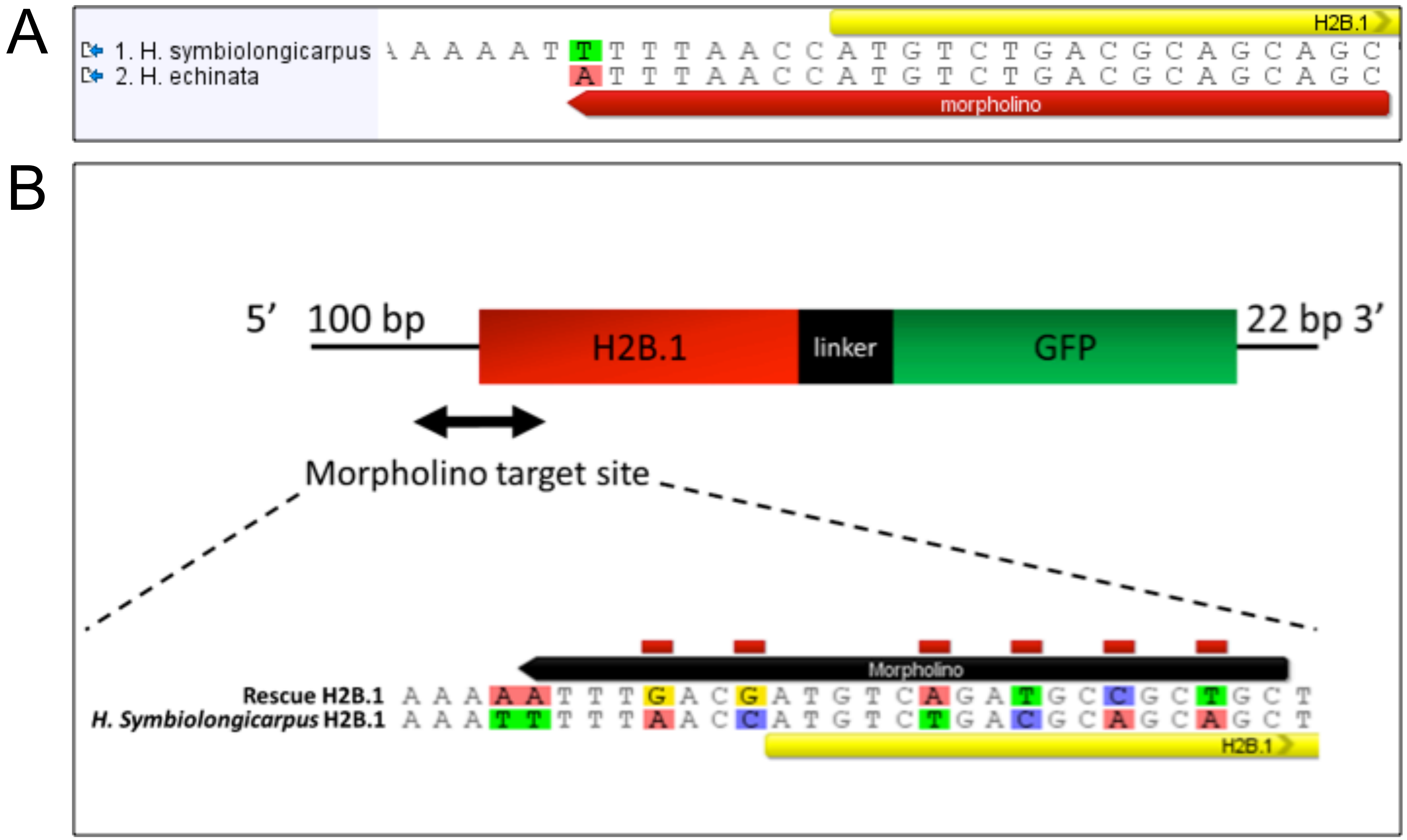

Fig. S1. Design of morpholinos. Alignment of the *H. echinata* H2B.1 morpholino with the H2B.1 sequence of *H. symbiolongicarpus* showing only 1 mismatch at 5' position. B. Design of H2B.1 rescue mRNA with mutated morpholino target site.

Development • Supplementary information

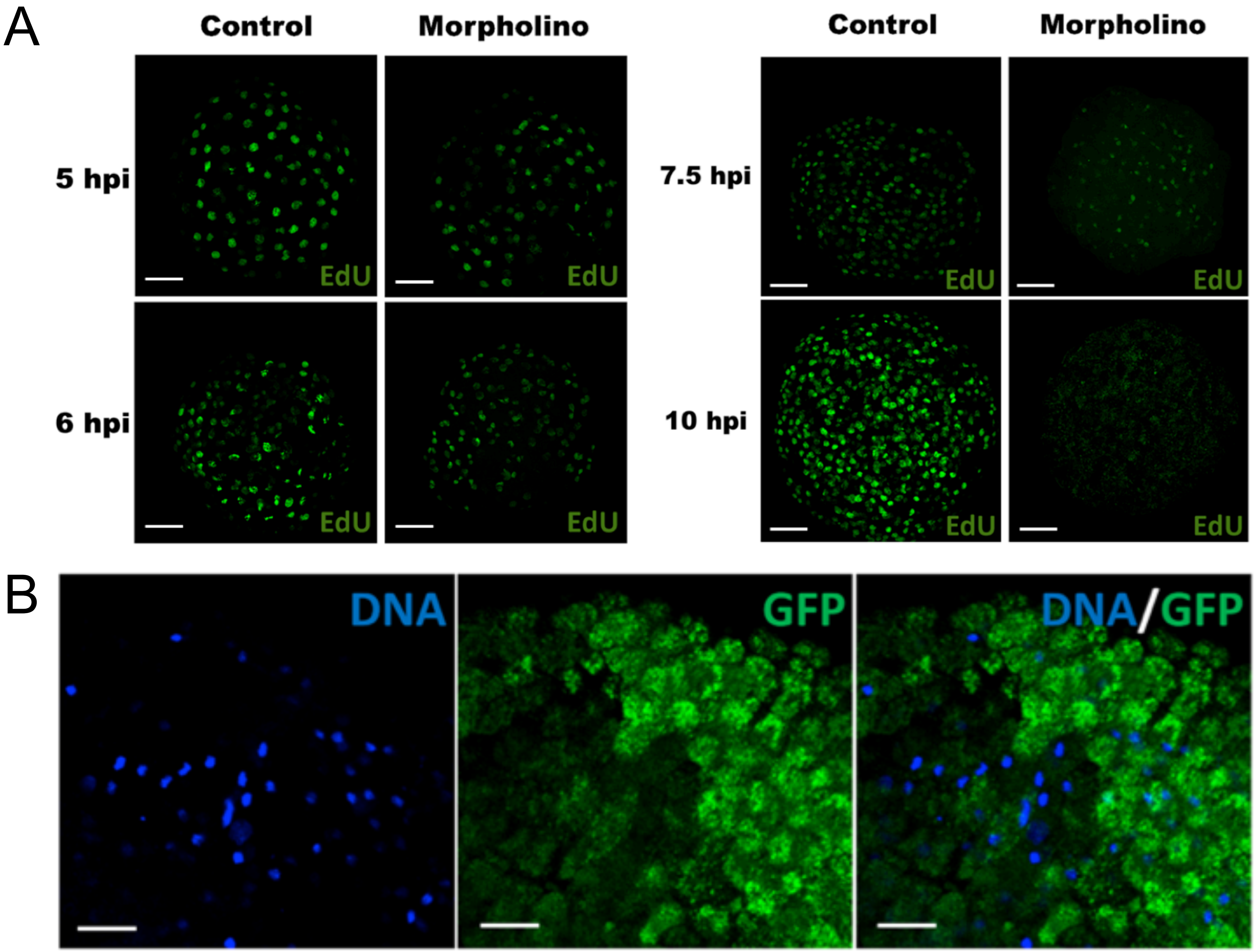

Fig. S2. Analysis of S phase cells after H2B.1 morpholino injection. A. Comparison of EdU incorporation at indicated hours post injection (hpi) with either non-specific control of H2B.1-specific morpholino. Scale bars 50 µm. B. GFP control expression in the cytoplasm during embryo development. Scale bars 25 µm.

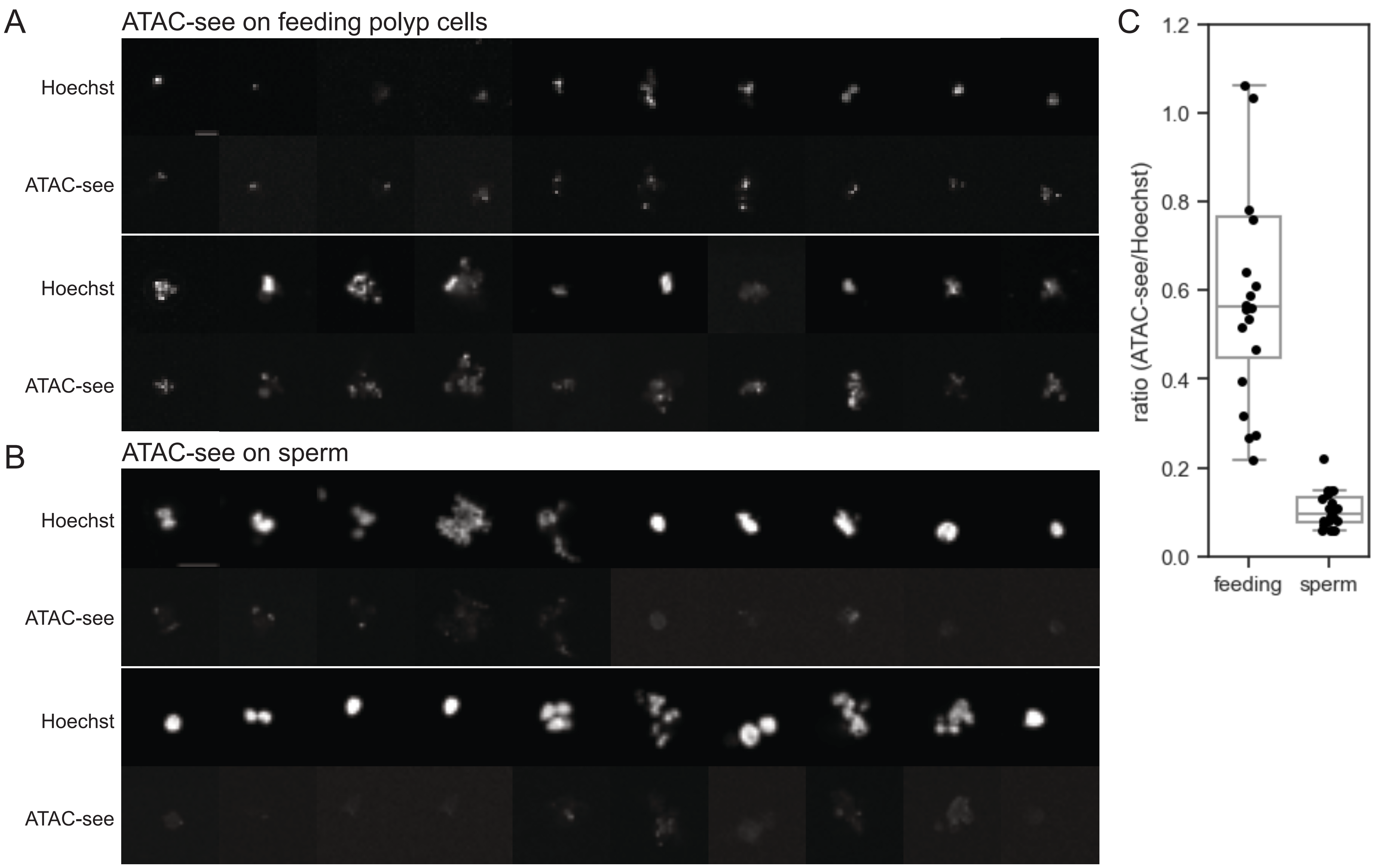

Fig. S3. ATAC-see of feeding polyp and sperm cells. A. Feeding polyp cells imaged for Hoechst 33258 staining of DNA (upper row) and ATAC-see (lower row) for 20 representative images at 40x magnification. Scale bar 10 μm. B. Imaging for sperm cells as in A at 100x magnification. C. Quantitation of ratio of total ATAC-see to Hoechst signal in images showing reduced ATAC-see for sperm under equivalent reaction conditions.

Table S1. Sequences of Hydractinia histone genes, proteins and promoter region.

>HeH2A gene
ATGTCTGGACGTGGAAAAGGTGGAAAAGCTAAGGCTAAAGCCAAGACAAGATCCTCAAGAGCTGGACTTCAAT
TTCCAGTCGGTAGAGTGCATAGATTCCTTCGTAGAGGGCACTATGCTAACCGAATTGGATCTGGAGCACCAGT
ATACTTAGCAGCCGTCTTGGAATATTTATCTGCTGAGATATTGGAGTTGGCTGGTAACGCAGCAAGAGACAAC
AAAAAAGCTAGAATTATCCCAAGACATTTACAATTGGCTGTTCGTAATGATGAAGAATTAAACAAACTTTTGA
GCGGTGTAACCATTGCAGCTGGTGGTGTTTTGCCAAACATTCAAGCTGTCTTACTTCCAAAGAAGAACGACAA
AGGACAGAAGAAGTAA

>HeH2B.1 gene
ATGTCTGACGCAGCAGCTAAAGGAGGAAAACAGGCACCTAAAGTAGCCAAGAAAGGTGAAAAGAGAGCCGGCA
AAAAAGGAGGAAAGATTGGTGGAACTGGTGAAAAGAAACGCAAGAAGAAGAGAAAGGAAAGTTATGCCATTTA
CATCTACAACGTTTTGAAACAAGTTCACCCAGATGTCGGAGTTTCTAGCAAAGCTATGAGCATCATGAACTCA
TTTGTCAACGACATCTTTGAGCGCATTGCTTCTGAAGCTTCGCGTTTGGCTCTTCAAAACAAAAAGTCGACCA
TCTCTTCTCGTGAAATTCAAACCGCAGTACGTCTTCTCTTGCCTGGAGAACTTGCAAAACACGCAGTCAGTGA
AGGAACAAAAGCCGTCACAAAATACACAAGCAGCAAGTAA

>HeH2B.3 gene
ATGGCTGGAAGTCCAAGAAAAGGAAGCCCAAAGAAAGCATCCTCTAGAGCTGCAAGCCCTAAAAGAGCTGCGA
GTCCTAAAAGAGGTGGAAGTCCAAAAAGAGGTGGAAGCCCAGCGAAAAAGGGAAAGGCGATTAAGAAAGCTGG
CAAGAGAAAAACAAACAAGAAAGCCACAACCAAGAGGCGAAGAAGTCGAAGGGAGAGTTATGGCATGTATATC
TACAAAGTTTTGAAACAAGTTCATCCTGATGTCGGAATTTCAAGCAAAGCGATGAGCATCATGAATTCTTTCG
TCAACGACATCTTTGAAAGATTAGCAGGCGAAGCTTCCAAACTTGCTCATCACAACAAACTACGAACCATCTC
GTCTCGTGAGGTACAAACTTCAGTTCGCTTGTTGTTGCCTGGTGAACTTGCGAAACACGCTGTGAGTGAAGGA
ACGAAAGCTGTTACCAAGTACACAAGCTCCAGATAA

>HeH2B.6 gene
ATGGCAAGTCCAAGAAAAGGAAGTCCAAGAAAAGGAAGTCCAAAGAAAGGGAGTCCAAAGAAGACATCCAGAG
CTGCTAGTCCGAAAAGGGGAAGCCCAAAGAAAGGGAAAGGCATGGCAGCCAAGAAAGGAGGTGTGAGAAAGGG
TGCAAAGAAAAAATGCAACAAAGAGACGAAGAAGCCGAAGAGAAAGTTATGGTATCTATATTTACAAAGTTTTG
AAACAAGTTCACCCTGATGTTGGAATTTCAAGTAAAGCTATGAATATCATGAATTCCTTTGTCAATGATATCT
TCGAAAGATTGGCTGGCGAGGCGTCGAGACTTGCTCATCACAACAAGAAACAAACCATCGCTTCTCGTGAAGT
CCAAACTTCAGTACGTTTGTTGCTTCCTGGTGAACTTGCAAAGCATGCAGTGAGCGAGGGAACAAAGGCTGTG
ACCAAATACACAAGTTCCAAGTAA

>HeH3 gene
ATGGCTCGTACAAAGCAAACTGCACGTAAATCTACTGGAGGAAAGGCTCCACGAAAACAACTCGCCACTAAAG
CTGCGAGAAAAAGCGCACCAGCTACTGGAGGAGTGAAAAAACCACATCGTTACAGACCTGGTACAGTTGCTCT
CAGAGAAATCAGAAGATACCAGAAGTCAACCGAGCTCTTGATCCGCAAGTTGCCTTTCCAGCGTCTTGTGCGA
GAAATTGCTCAGGACTTCAAAACAGATCTGCGATTCCAGAGCACAGCCGTTATGGCTCTGCAGGAGGCTTCTG
AAGCGTACCTTGTTGGCTTATTCGAAGATACTAACTTGTGTGCCATTCACGCAAAACGAGTTACTATCATGCC
TAAAGACATCCAGTTGGCAAGAAGAATTCGTGGTGAACGAGCATAA

>HeH4 gene
ATGTCTGGTCGTGGTAAAGGTGGCAAAGGTCTGGGTAAAGGTGGTGCGAAACGTCATCGTAAAATCCTGCGCG
ATAACATTCAGGGCATTACCAAACCAGCCATCCGTCGTTTGGCTCGCCGCGGCGGCGTTAAGCGTATCTCGGG
CCTGATCTATGAAGAGACTCGCGGCGTACTGAAGGTGTTCCTTGAAAATGTAATCCGTGACGCGGTTACCTAT
ACTGAACACGCGAAACGAAAAACCGTTACGGCAATGGACGTCGTGTACGCTCTCAAACGTCAGGGCCGTACAC
TGTACGGGTTCGGTGGTTAA

>HeH2A protein
MSGRGKGGKAKAKAKTRSSRAGLQFPVGRVHRFLRRGHYANRIGSGAPVYLAAVLEYLSAEILELAGNAARDN
KKARIIPRHLQLAVRNDEELNKLLSGVTIAAGGVLPNIQAVLLPKKNDKGQKK

Development • Supplementary information

>H2B.1 protein
MSDAAAKGGKQAPKVAKKGEKRAGKKGGKIGGTGEKKRKKKRKESYAIYIYNVLKQVHPDVGVSSKAMSIMNS
FVNDIFERIASEASRLALQNKKSTISSREIQTAVRLLLPGELAKHAVSEGTKAVTKYTSSK

>HeH2B.3 protein
MAGSPRKGSPKKASSRAASPKRAASPKRGGSPKRGGSPAKKGKAIKKAGKRKTNKKATTKRRRSRRESYGMYI
YKVLKQVHPDVGISSKAMSIMNSFVNDIFERLAGEASKLAHHNKLRTISSREVQTSVRLLLPGELAKHAVSEG
TKAVTKYTSSR

>HeH2B.6 protein
MASPRKGSPRKGSPKKGSPKKTSRAASPKRGSPKKGKGMAAKKGGVRKGAKKNATKRRRSRRESYGIYIYKVL
KQVHPDVGISSKAMNIMNSFVNDIFERLAGEASRLAHHNKKQTIASREVQTSVRLLLPGELAKHAVSEGTKAV
TKYTSSK

>HeH3 protein
MARTKQTARKSTGGKAPRKQLATKAARKSAPATGGVKKPHRYRPGTVALREIRRYQKSTELLIRKLPFQRLVR
EIAQDFKTDLRFQSTAVMALQEASEAYLVGLFEDTNLCAIHAKRVTIMPKDIQLARRIRGERA

>HeH4 protein
MSGRGKGGKGLGKGGAKRHRKILRDNIQGITKPAIRRLARRGGVKRISGLIYEETRGVLKVFLENVIRDAVTY
TEHAKRKTVTAMDVVYALKRQGRTLYGFGG

>Hydractinia Piwi promoter 2080bp
CAGATGATCCGCAGACAATAGACCTTTTTGAATGTTTACATTTTCCGCGGTAAGCCCTGTCAGCTTTAACAAT
TGCATGGCAGGCAAAAAGATGCGGGTGCTAGGTAAAGTGAGGTCCTTTTTAGTCTGTTTTATTATTACCACTT
TATATGAATATATGTCAATCAAAAGTTATCTATCTACACTCGATAATAGTTTAAAGCCTCAAAAATGTTTCCG
TAGAAAGAAATGGGCTGAAAAAGACCTTTCTTCAGCAAGCTTCCGCATCTTTCTGCCTGCCACGCAGTTGTTA
GAGCCAGAAGGGCTACACGAAAATGTAAACATTTAAAAAGGTCTATAGACATGTTTAATTTGCCCTAATTTTT
GGTTGGCAATAGAATGAATATATATATAAGGAATAATGAAATTTTCCCCCATGTATTATTTAATATGTCTAGG
ACAAAAAAGTCATGTGACTTGGCTTTTAGCCAATAAGATTGTTGTAATTTTTAGGGGGAAAACACTGTGGCAA
AATGTTCCTATAACACTTTTATATGCAACTCATATCATAAATTCAGGAGAGTGTGTATTGTTTGCCCCAAATG
TGATGTAATCATTGCCACACAAGAATACAATATCTAGCTGGCAGGATCCCTTGTTTGCTGCTTGACCTCAACT
GTTATATGTGGATGTTTGCAGATTATCAGCACCCTTGTCTCATAATGAAAAGTGAAAAAATCCCCGCCAACCC
TCTAGATAGATAAGGAATACATGAATTCACAGTTTCAGCTAACTATTGTCTAAATAAATATTTCTTTTGTTAC
AATGCTTTTTTTGAGAATTTAAATAAAGGAAGTGAACTGTTATCAAGAAGGCTCTGGCCATGAAGAAAAGTGA
AATAATTTTTTTGTGAAATATCTTGAAAAAAAAAGTTCAAATTCCTTCACATTGTTTTAAGTAGGGTTACTGC
CAATCTATGAAGATTGGTCAGTGGTCCCTCGCTTTACATGAAAAAATAGCCAGACTTCAACGCCAAGTATAAA
CCACTCTGCCTGCTACACATATAGGCTATTCACCTAGATATAGTCATGCAGCCTTGGCAGCGATTCAATATGG
GCAATTGAATATTTAGCATAGCCCCCGGGTTAACTCAAGCCATGTGAGAGAATTGGGGAGATAGCGCACTGTG
TGGTGGGCCGTTGGATGTTGCAGGGAGTTGTCTGGCAGAGCGCTGACCCTAATTGAGTTTACGTAGCTCAAAG
AGAGCATTAAATACTCCAGGATAGATAGATAGATAGATCTACTATCTATCTATCTAGCTCAAAGAGAGCATTA
AATACTCCAGGACTCCCCATCAAAGCCATGACCCCTCCTGGAAATAATAGACAGGGTATATCCCTCGATATTT
GTGAGGCTAGCCATGTAAAATATGCACATCTATCTATCTATTACATGATAACTGGTCTGGTCTTGGTACACAT
GCATCACCACTTTCCCTACCATGTTTTATTTTGTGATGCTGCGCCAAAGTATATCACAATGGTTGTATTTGTT
CAAATTCAGCGCCCCTTTAGCTTACGGTGGATTTTCACTTTGATTTCAGTGAGGAACAGAAATGTGCAAGGTT
ACCGCGTTATATGATATTTATTTTTTATTCATTTTTTTTATCCCATCCTTTTTTAACTTCTAGCACAAAATAA
TTTTTAGTCTCTTTAAAAATTATCGCACATTAAATGTTGAATTAAAGATTCATAAAAACTTTTTTTTTAGTGT
GATAATTTATATTATATACCACAAGTTTTGTTTGTTGTCCACTTATTGACTAAAGTCATAATAGTCACTCGGA
ATATGACTAAAATTAAAATAAATGCATGTTTTCTTATTGTCGTATTTTCAAAAAATATAAATTTTTATCTGTC
TCCCCTCCTATTCTTTTCCTTTCAAACAAAACTTGACAAGTGGAAACCCACCCTTGTGTGAAGCAAGGTTGGT
TTCTGTTTGGTGCATTATTAGACCATCACTGAACTTCTTCTCAACATTTATGAATAAAATAACGTATTTTGAG
TCATATTTAATAATTACGAAAATTTTCATTACATTT

Development • Supplementary information

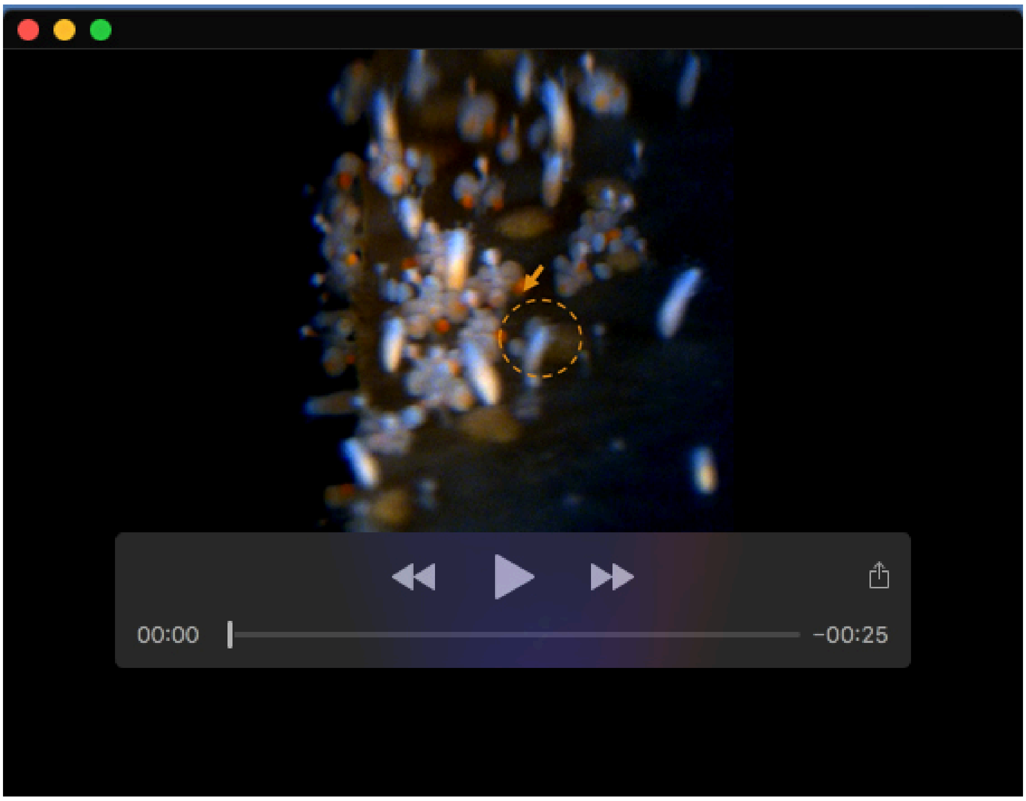

Movie 1. Spawning of Hydractinia female colony. Orange arrow indicates female sexual polyp suspended on vertical surface. Orange circle highlights release and slow sedimentation of unfertilised eggs in real time. Width of region in movie frame is 1.5 cm

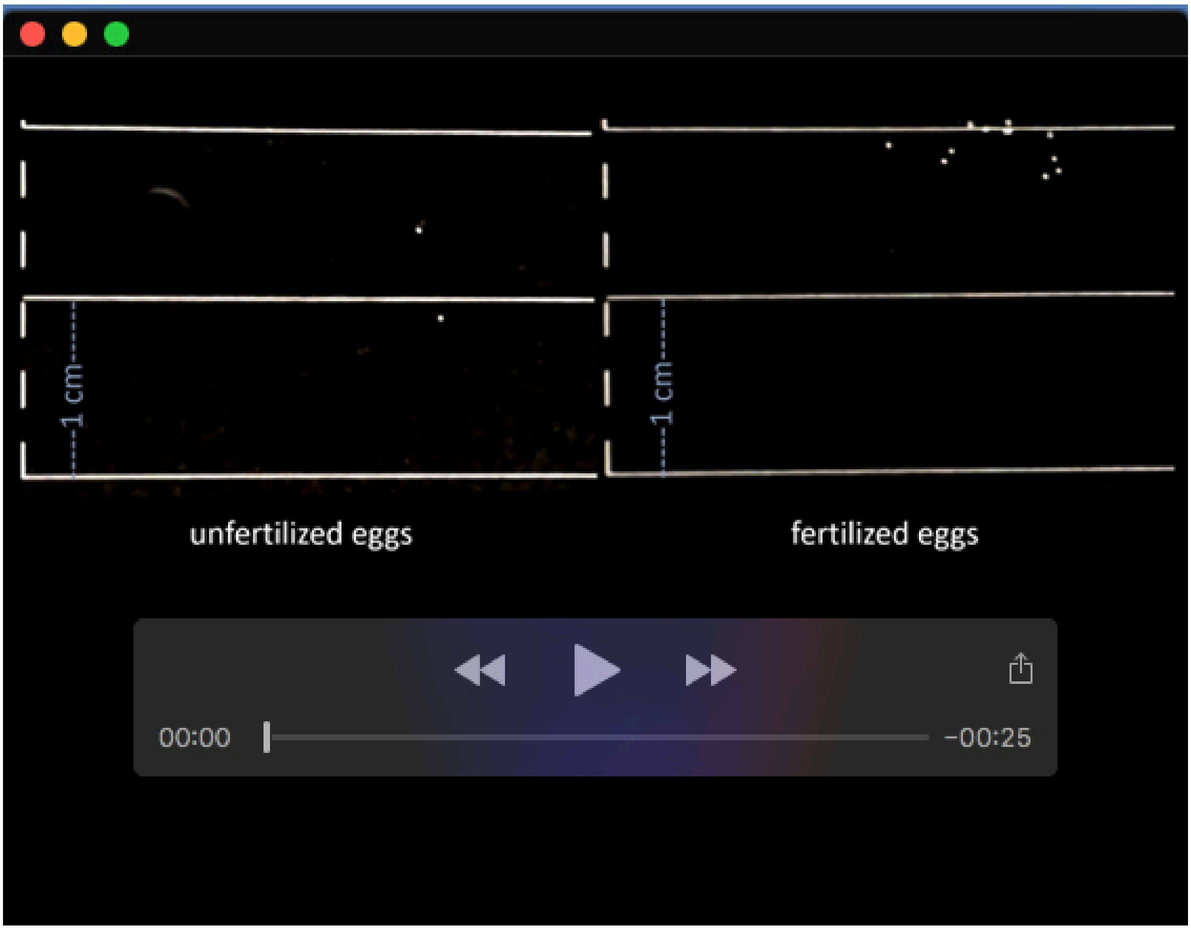

Movie 2. Hydractinia eggs are more buoyant than zygotes. Unfertilised egg (left) and fertilised zygote (right) sedimentation in real time through a 1 cm window.
