# Supplementary material for: Hydrozoan sperm-specific SPKK motif-containing histone H2B variants stabilise chromatin with limited compaction
Source: Development. 2023 Jan 12;150(1):dev201058. doi: 10.1242/dev.201058 (PMC9903204; doi:10.1242/dev.201058)
Supplement: Supplementary file 1 [file develop-150-201058_review_history.pdf]

Hydrozoan sperm-specific SPKK motif-containing histone H2B variants stabilise chromatin with limited compaction

Anna Török, Martin J. G. Browne, Jordina C. Vilar, Indu Patwal, Timothy Q. DuBuc, Febrimarsa, Erwan Atcheson, Uri Frank, Sebastian G. Gornik and Andrew Flaus
DOI: 10.1242/dev.201058

Editor: Maria Elena Torres-Padilla

Review timeline

Original submission:	2 September 2021
Editorial decision:	3 October 2021
Resubmission:	21 July 2022
Editorial decision:	10 August 2022
Second revision:	28 November 2022
Accepted:	2 December 2022

Original submission

First decision letter

MS ID#: DEVELOP/2021/200152

MS TITLE: Hydrozoan sperm-specific H2B histone variants stabilize chromatin and block transcription without enhancing chromatin condensation

AUTHORS: Anna Torok, Martin JG Browne, Jordina C Vilar, Indu Patwal, Timothy Q DuBuc, F Febrimarsa, Erwan Atcheson, Andrew Flaus, Uri Frank, and Sebastian G Gornik
ARTICLE TYPE: Research Report

Dear Dr. Gornik,

I have now received all the referees reports on the above manuscript. The referees' comments are appended below, or you can access them online: please go to BenchPress and click on the 'Manuscripts with Decisions' queue in the Author Area.

As you will see from their reports, the referees recognise the potential of your work, but they also raise significant concerns about it. Given the nature of these concerns, I am afraid I have little choice other than to reject the paper at this stage. In particular, all the reviewers, as well as the editor, express that your work is of high potential interest given the scarce literature on histone variants outside of major classical models. However, overall the reviewers expressed that the advance offered is incremental considering your earlier work on the identification of the male gonad-specific H2B variant, spH2Bs, in hydrozoan cnidarian and that there are several concerns with either data quality or over-interpretation. Considering the reviewers' reports, it would seem that the manuscript at this point appears preliminary and the overall conclusions do not seem fully supported by the data due to interpretation or lack of side-by-side suitable controls.

However, having evaluated the paper, I do recognise the potential importance of this work. I would therefore be prepared to consider as a new submission an extension of this study that contains new experiments, data and discussions and that address fully the major concerns of the referees. The work required goes beyond a standard revision of the paper. Please bear in mind that the referees (who may be different from the present reviewers) will assess the novelty of your work in the

© 2023. Published by The Company of Biologists under the terms of the Creative Commons Attribution License (https://creativecommons.org/licenses/by/4.0/).

context of all previous publications, including those published between now and the time of resubmission.

<u>Reviewer 1</u>

Advance summary and potential significance to field

The authors had previously identified the male gonad-specific H2B variant, spH2Bs, in hydrozoan cnidarian (Hydractinia echinate). Here they report the biochemical and functional characterization of spH2Bs in vitro and in vivo, using the model of H. symbiolongicarpus.
The methodology chosen to investigate the properties of spH2Bs are not always appropriate and the quality of the data presented is poor. Therefore, the data presented do not clearly support the authors' conclusions.

Comments for the author

Specific points are presented below.

1 - Figure 2D, the authors used native PAGE to analyze nucleosome arrays assembled on different DNA fragments containing either H2B.1, H2B.3 or H2B.6, to conclude on the ability of spH2Bs to mediate a significant chromatin compaction. However, this is a very rough and unsensitive assay to measure chromatin compaction and would not allow the authors to draw any conclusion on the role of spH2Bs in chromatin compaction. Additionally, a control corresponding to compact chromatin is missing.

2 - The design of the experiment and the quality of the data showing the pattern of chromatin digestion by MNase (Figure 3B) are very poor.
Indeed, to show a differential chromatin digestion by MNase it is necessary to present the kinetics of chromatin digestion by MNase, not only a single point as shown in Figure 3B.

3 - The results of the ATAC-see experiment shown here is not conclusive. It seems that in progenitor cells, in a number of cases, the signal is outside of the nucleus. It seems that the authors are comparing a background signal in progenitor cells with no-background in mature sperms. It is not clear if the Tn5 transposase has properly worked.

4 - The authors performed ApoI restriction enzyme digestion on oligonucleosomal arrays assembled in vitro on a 2080 bp Hydractinia DNA fragment with H2B.1, H2B.3, or H2B.6 containing histone octamers (Fig. 3D).
First, it is not clear why the authors mention that they have performed NATIVE PAGE. Usually the term "native" is used in the case of nucleoprotein complexes but not purified DNA fragments. Here it is not clear what the authors have loaded.
Second, in the digestion profiles shown, the only major difference is the point 3 min, but since this point is under-loaded in H2B.6, it is difficult to conclude.

5 - It is also difficult to draw a clear conclusion from the data shown in Figure 4. Indeed, controls are lacking to make sure that the authors have injected exactly the same amounts of the rescuing mRNAs and that all parameters remain comparable when they inject different H2B encodings mRNAs.

Minor points:

6 - Lane 43: Our knowledge of chromatin transformations and remodeling in spermatogenic cells has evolved tremendously since 1991 and 2002. It is not clear why the authors are citing these old reviews in support of their statements on chromatin remodeling during spermatogenesis.

7 - In contrast to the authors' statement (lane 44), SNBPs do not replace only somatic histones but in many species also testis-specific histone variants expressed in earlier stages.

8 - Lane 593, the authors of the cited reference are missing

© 2023. Published by The Company of Biologists under the terms of the Creative Commons Attribution License (https://creativecommons.org/licenses/by/4.0/).

Reviewer 2

Advance summary and potential significance to field

The Authors report evolution in Cnidaria of a sperm-specific family of H2B variants- spH2Bs- characterized by KSPKK repeats in the N-terminal tail. These variants do not cause overall reduction of sperm nucleus size but do confer thermostability in vitro and reduce chromatin according to in vitro assays. Reduction of chromatin accessibility is also observed in mature sperm compared to somatic and sperm progenitor cells. To assay more directly the functions of spH2Bs, the authors inject spH2B-GFP mRNAs in blastomeres of early embryos. This has no major effect on embryo development. The Author then block H2B1 - the major type of H2B variant) using morpholino. This causes embryo arrest and reduced RNA synthesis (measured by uridine incorporation) that is rescued by morpholino resistant H2B-1 but not by spH2Bs, suggesting a distinct function of spH2Bs. Overall the data is interesting because there is such scarce literature on histone variants outside of the major classical model.

Comments for the author

The report would profit from more cautious conclusions that reflect the results and do not over interpret them.

Major:
A major problem with the manuscript is the interpretation of experiments reported in Fig4. The Authors show that spH2Bs do not rescue the defects caused by the reduction of H2B1. However, this does not allow to conclude that H2B block transcription and cell cycle. First it is not showed that the embryo arrest is the direct consequence of cell cycle problems or transcription. Further we have little information regarding the amount of proteins and their localization of H2B-GFP in arrested embryos.

Minor:
Fig.2 Expression pattern of H2B1 in sperm progenitor cells is missing. Although published , it would be useful to include here as a control.

Fig3A y axis please correct typo "fluoresence"

The comments related to Fig3B and C are correct but they are placed in a section titled "SP[K/R][K/R] motifs stabilise chromatin structure and restrict chromatin accessibility". The title implies that the authors test directly the function of the KSPKK motif in their in vitro and in vivo assays. But this is not the case . So please use a title that strictly reflects the findings : Chromatin accessibility is reduced in sperm and by spH2Bs according to in vitro assays.

Discussion
"Hydractinia spH2Bs do not appear to increase chromatin density." There is not data on cnidarian sperm density here and even less data on spH2B mutant sperm density, which would be required to reach such a conclusion. Please modify or remove.

The general argument in the discussion does not address the findings of the paper directly.

It would be better for example to dig deeper in the evolution of KSPK H2Bs in sea urchins and cnidarians. The role of the motif more generally in histone variants.

On a similar line the title and abstract should be more conservative and avoid overstatements

Reviewer 3

Advance summary and potential significance to field

Török et al. explore the functional properties of an hydrozoan-specific histone variants associated to sperm cells.

© 2023. Published by The Company of Biologists under the terms of the Creative Commons Attribution License (https://creativecommons.org/licenses/by/4.0/).

Through in vivo and (thorough) in vitro assays, the authors argue that these spH2B variants reduce DNA accessibility (and transcription) without directly affecting global chromatin compaction. This work represents an extension of the results reported by Török et al. 2016. Except for a few specific points, I find the data convincing and the findings and their discussion really interesting.

Comments for the author

Major comments:
1. The authors should show that "H2B.1 is concomitantly downregulated" (line 92). This is central to the point that an H2B variant replacement exists (even more if no nuclear compaction is observed).

2. The authors should discuss/clarify how, in comparison, protamines would be expected to behave in the in vitro assays such as the ones described in lines 103-114. Perhaps even use protamines as a positive control.

3. Fig. 2B is inadequate: samples should be run in the same gel or at least the internal gel ladder should be shown (not a manually drawn ladder). I also find the assay in Fig. 3C rather unconvincing, given that Tn5 tagmentation conditions can vary greatly, so two pictures seem skim evidence of reduced accessibility cause by spH2B histones (much less that they are the mechanisms directly responsible for this possible reduced accessibility). I must say, though, that the ApoI assay does seem to convincingly point at reduced accessibility.

4. It would be interesting to quantify by RNA-seq the transcriptional effects cause by the spH2B mRNA / H2B.1 morpholino co-injection. E.g. to see if these effects are genome-wide or in particular loci. In addition, the authors could inject an spH2B mRNA with mutated SPKK motif residues to test the specificity of this aa motif in causing transcriptional silencing.

Minor comments:
- Figure 1A is very confusing. Shouldn't "Histone-type" proteins should appear everywhere?
- Are SPKK motifs found in other hydrozoan histones? (e.g. in H1 in sea urching).
- Please make sure to include details on all the experimental methods (instead of referring to Török et al. 2016 publication).

First revision

Author response to reviewers' comments

Reviewer 1

Advance Summary and Potential Significance to Field:

The authors had previously identified the male gonad-specific H2B variant, spH2Bs, in hydrozoan cnidarian (Hydractinia echinate). Here they report the biochemical and functional characterization of spH2Bs in vitro and in vivo, using the model of H. symbiolongicarpus.

The methodology chosen to investigate the properties of spH2Bs are not always appropriate and the quality of the data presented is poor. Therefore, the data presented do not clearly support the authors' conclusions.

We thank the reviewer for the constructive comments. We are very grateful for the efforts of all reviewers to provide detailed feedback.

We wish to point out that histones are a multigene family of highly expressed essential proteins and their contribution to bulk chromatin characteristics has been only characterised directly in a few organisms using detailed and optimised methods often based on deep sequencing, high resolution microscopy and single molecule analysis. These recent technological methods were preceded by classical approaches involving in vitro biochemistry and imaging of

© 2023. Published by The Company of Biologists under the terms of the Creative Commons Attribution License (https://creativecommons.org/licenses/by/4.0/).

nuclear properties. We would like to suggest that exploring a wider range of metazoans can give insights into the diversity of chromatin properties, but that advanced methods are not yet established or optimised for these systems so classical approaches can also be appropriate, especially when they align with earlier work such as on sea urchin spH2Bs.

We have significantly revised the presentation of data and the conclusions based on the feedback from all the reviewers. We wish to underline our sincere appreciation to all reviewers for their interest and inputs, and we have tried to address all points in detail

Reviewer 1 Comments for the Author:

Major 1

Figure 2D, the authors used native PAGE to analyze nucleosome arrays assembled on different DNA fragments containing either H2B.1, H2B.3 or H2B.6, to conclude on the ability of spH2Bs to mediate a significant chromatin compaction. However, this is a very rough and unsensitive assay to measure chromatin compaction and would not allow the authors to draw any conclusion on the role of spH2Bs in chromatin compaction. Additionally, a control corresponding to compact chromatin is missing.

Due to the space limitations of the article we were unable to provide a full explanation of the in vitro assay for compaction. We do not have ready access to either analytical ultracentrifugation (AUC), atomic force microscopy (AFM) or optical tweezers which are common quantitative methods for compaction in established models and comprise whole studies in their own right.

Instead, we have used an classical native gel electrophoresis approach with a mixed agarose-polyacrylamide system (PMID 15567867) which is well-correlated with AUC and highly sensitive to composition and compaction (PMID 7673242, 12614610, 15644197). In fact, native gel electrophoresis has been used to demonstrate compaction of nucleosome arrays on the same 5S repeats by polymeric SPKK peptides (PMID 9033394).

The most common approach to induce compaction by addition of Mg^{2+} in samples greatly distorts native gels with low ionic strength buffers so we were unable to include a positive control for compaction. Unfortunately, we were unaware of the SPKK peptide publication at the time the experiments were carried out and we are no longer in a position to repeat this complicated experiment with a SPKK peptide control.

Based on the cited precedents, we suggest that the equivalence of native gel migration is evidence of similar compaction at the resolution and under the conditions described. We hope that these precedents, replication using 3 different arrays, and consistency with ATAC- see will reassure the reviewer and readers. We have improved the text and added citations.

Major 2

The design of the experiment and the quality of the data showing the pattern of chromatin digestion by MNase (Figure 3B) are very poor. Indeed, to show a differential chromatin digestion by MNase it is necessary to present the kinetics of chromatin digestion by MNase, not only a single point as shown in Figure 3B.

MNase digestion of bulk chromatin is a sampling of the nucleosomal repeat length at multiple points along native chromatin. This is the basis of the classic comparison by Widom (PMID 1736292) using a tabulation of single point digestions by van Holde (Chromatin, 1989). A time course of digestion results in the nucleosome core particle (PMID 4825889).

We have updated the text to clarify that the increased repeat length does not imply reduced accessibility. We agree with the reviewer that a kinetic analysis would be necessary to demonstrate extended protection around the nucleosome core particle. This point is addressed in figures 3D and 3E.

© 2023. Published by The Company of Biologists under the terms of the Creative Commons Attribution License (https://creativecommons.org/licenses/by/4.0/).

We also agree that the submitted figure 3B was difficult to interpret so we have expanded the panel to include the ladder lanes and reported the observed nucleosomal repeat length. Interestingly, this shows the same increase of 20 bp reported by Green and Poccia (1988) for sea urchin.

Major 3

The results of the ATAC-see experiment shown here is not conclusive. It seems that in progenitor cells, in a number of cases, the signal is outside of the nucleus. It seems that the authors are comparing a background signal in progenitor cells with no-background in mature sperms. It is not clear if the Tn5 transposase has properly worked.

We agree that the submitted figure 3C was difficult to interpret so we have substituted this panel with a parallel experiment we also performed comparing somatic and sperm cells. This shows Tn5 transposase accessibility in multiple regions of the Hoechst-stained somatic nuclei but not for the sperm cells. Importantly, sperm cells have Tn5 staining at sites adjacent to the nuclear DNA which we interpret as mitochondria. This provides an internal positive control demonstrating the Tn5 transposase activity.

Major 4

The authors performed ApoI restriction enzyme digestion on oligonucleosomal arrays assembled in vitro on a 2080 bp Hydractinia DNA fragment with H2B.1, H2B.3, or H2B.6 containing histone octamers (Fig. 3D). First, it is not clear why the authors mention that they have performed NATIVE PAGE. Usually the term “native” is used in the case of nucleoprotein complexes but not purified DNA fragments. Here it is not clear what the authors have loaded. Second, in the digestion profiles shown, the only major difference is the point 3 min, but since this point is under-loaded in H2B.6, it is difficult to conclude.

The legend to figure 3E referred to PAGE separation of DNA fragments. This was labelled as native PAGE to avoid confusion with denaturing PAGE of single stranded DNA. We apologise that this confused the reviewer so we have changed this to “non-denaturing” in the legend and methods for clarity.

Figure 3E plots the point of median migration of band intensities at each time point. This incorporates the integrated information from all bands and is normalised for each lane to remove the effect of differential loading such as in fig 3D H2B.6 3 min digestion. We agree with the reviewer that plotting individual bands as in figure 3D were less representative so these have been removed. The plot in fig 3E combines data from two H2B.3 replicates and the H2B.6 array observations in fig 3D, each performed in parallel with H2B.1 arrays. This demonstrates the reduced accessibility of ApoI in the spH2B-containing arrays relative to H2B.1 arrays. We have improved the explanation in the legend and methods.

Major 5

It is also difficult to draw a clear conclusion from the data shown in Figure 4. Indeed, controls are lacking to make sure that the authors have injected exactly the same amounts of the rescuing mRNAs and that all parameters remain comparable when they inject different H2B encodings mRNAs.

Fig 4 panels I and N show quantitative summaries of multiple embryos. We have added labels within the panels and improved the explanation in the methods to state the uniform concentrations and volumes that were used in our protocol for microinjections.

Minor 6

Line 43: Our knowledge of chromatin transformations and remodeling in spermatogenic cells has evolved tremendously since 1991 and 2002. It is not clear why the authors are citing these old reviews in support of their statements on chromatin remodeling during spermatogenesis.

© 2023. Published by The Company of Biologists under the terms of the Creative Commons Attribution License (https://creativecommons.org/licenses/by/4.0/).

We have carefully selected and updated the references, within the limits of the manuscript format.

Minor 7

In contrast to the authors' statement (line 44), SNBPs do not replace only somatic histones but in many species also testis-specific histone variants expressed in earlier stages.

This is an important point. We have updated the text to read "This is achieved, in part, by sperm nuclear basic proteins (SNBPs) that replace canonical and other variant histones."

Minor 8

Line 593, the authors of the cited reference are missing

We have corrected this oversight.

Reviewer 2

Advance Summary and Potential Significance to Field:

The Authors report evolution in Cnidaria of a sperm-specific family of H2B variants- spH2Bs- characterized by KSPKK repeats in the N-terminal tail. These variants do not cause overall reduction of sperm nucleus size but do confer thermostability in vitro and reduce chromatin according to in vitro assays. Reduction of chromatin accessibility is also observed in mature sperm compared to somatic and sperm progenitor cells. To assay more directly the functions of spH2Bs, the authors inject spH2B-GFP mRNAs in blastomeres of early embryos. This has no major effect on embryo development. The Author then block H2B1 – the major type of H2B variant) using morpholino. This causes embryo arrest and reduced RNA synthesis (measured by uridine incorporation) that is rescued by morpholino resistant H2B-1 but not by spH2Bs, suggesting a distinct function of spH2Bs. Overall the data is interesting because there is such scarce literature on histone variants outside of the major classical model.

The report would profit from more cautious conclusions that reflect the results and do not over interpret them.

We thank the reviewer for this positive interest, and we are very grateful for the efforts of all reviewers in providing detailed feedback that has allowed us to improve this work. We have significantly revised the conclusions based on the feedback from all reviewers.

Reviewer 2 Comments for the Author:

Major 1

A major problem with the manuscript is the interpretation of experiments reported in Fig4. The Authors show that spH2Bs do not rescue the defects caused by the reduction of H2B1. However, this does not allow to conclude that H2B block transcription and cell cycle. First it is not showed that the embryo arrest is the direct consequence of cell cycle problems or transcription. Further we have little information regarding the amount of proteins and their localization of H2B-GFP in arrested embryos.

We have revised the conclusions of this experiment to state that spH2B incorporation in chromatin in place of H2B.1 leads to cell cycle arrest and transcriptional block. The injected mRNAs are translated as demonstrated by nuclear GFP localisation.

© 2023. Published by The Company of Biologists under the terms of the Creative Commons Attribution License (https://creativecommons.org/licenses/by/4.0/).

Minor 2

Fig.2 Expression pattern of H2B1 in sperm progenitor cells is missing. Although published, it would be useful to include here as a control.

We have updated the text to explicitly state the expression pattern in the Introduction.

Minor 3

Fig3A y axis please correct typo “fluoresence”

Figure 3 has been substantially improved and this has been corrected.

Minor 4

The comments related to Fig3B and C are correct but they are placed in a section titled “SP[K/R][K/R] motifs stabilise chromatin structure and restrict chromatin accessibility“.

The title implies that the authors test directly the function of the KSPKK motif in their in vitro and in vivo assays. But this is not the case . So please use a title that strictly reflects the findings : Chromatin accessibility is reduced in sperm and by spH2Bs according to in vitro assays.

We thank the reviewer for the suggested clarification and have updated the subtitle to “Chromatin accessibility is reduced in sperm and in vitro assays of spH2B-containing chromatin.”

Discussion

“Hydractinia spH2Bs do not appear to increase chromatin density.” There is not data on cnidarian sperm density here and even less data on spH2B mutant sperm density, which would be required to reach such a conclusion. Please modify or remove.

We observed only a small reduction in nuclear areas in figure 2C and no evidence of compaction on 3 different DNA arrays in vitro in figures 2D-E. However, we accept that the summary statement quoted from the Discussion is strong so we have reworded it to: “Our observations do not show evidence for Hydractinia spH2Bs significantly increasing chromatin compaction in assays either in vivo or in vitro.”

The general argument in the discussion does not address the findings of the paper directly.

We understand the reviewer’s wish for more narrative on the results. However, we are constrained by the short format of this article layout so we attempted to summarise the fining in two concise paragraphs. Principally, we are enthusiastic to explain to readers of Development how the detailed observations of chromatin can be related to the developmental challenges of hydrozoan cnidarians. We have modified the text to make the statement of findings clearer and to link the contextual points with the potential implications for Hydractinia sperm function.

It would be better for example to dig deeper in the evolution of KSPK H2Bs in sea urchins and cnidarians. The role of the motif more generally in histone variants.

We have included this point and cited our recent survey of SNBP evolution (Torok and Gornik, 2018).

On a similar line the title and abstract should be more conservative and avoid overstatements

We have significantly revised the title, abstract and conclusions based on the feedback from all reviewers.

© 2023. Published by The Company of Biologists under the terms of the Creative Commons Attribution License (https://creativecommons.org/licenses/by/4.0/).

Reviewer 3

Advance Summary and Potential Significance to Field:

Török et al. explore the functional properties of an hydrozoan-specific histone variants associated to sperm cells. Through in vivo and (thorough) in vitro assays, the authors argue that these spH2B variants reduce DNA accessibility (and transcription) without directly affecting global chromatin compaction. This work represents an extension of the results reported by Török et al. 2016. Except for a few specific points, I find the data convincing and the findings and their discussion really interesting.

We thank the reviewer for these positive comments. We are very grateful for the efforts of all reviewers in providing detailed feedback that has allowed us to improve the manuscript.

Reviewer 3 Comments for the Author:

Major 1.

The authors should show that “H2B.1 is concomitantly downregulated” (line 2). This is central to the point that an H2B variant replacement exists (even more if no nuclear compaction is observed).

We agree that the replacement of H2B.1 by spH2Bs during spermatogenesis is a central to our study. This was already shown in our previous publication by Torok et al (2016) so we have made sure that the citation of this important point is made explicitly.

Major 2.

The authors should discuss/clarify how, in comparison, protamines would be expected to behave in the in vitro assays such as the ones described in lines 103-114. Perhaps even use protamines as a positive control.

Protamines are estimated to drive compaction of DNA in mouse sperm by 40 fold compared to somatic interphase nuclei [PMID 938720, 7297606] and produce chromatin that is 6 fold more compact than mitotic chromosomes [PMID 2043729] as recently reviewed by Okada [PMID 35344802].

This is because the protamines drive toroidal DNA arrangement with both tight local and long-range packaging characteristics that approaches crystalline nature [PMID 2043729, 32392345]. In vitro protamine compacted toroids can have a radius as small as 10 nm [PMID 32392345] and the interhelical spacing of small toroids can be <3 nm [PMID 33784405], implying our ~2 kbp DNA arrays could form a cylinder of 20 nm diameter and 30 nm height with volume of ~1 x 10^4 nm^3. In contrast, the same length of DNA packaged as nucleosomes in a “30 nm fibre” arrangement would have 30 nm diameter and 22 nm height resulting in ~50% larger volume of ~1.5 x 10^4 nm^3. Predicting native gel mobility is difficult since protamines are likely to provide more charge neutralisation per unit length of DNA bound than histones. We also note that Hydractinia genomes do not encode detectable protamines or other SNBPs. Unfortunately, we are no longer in a position to repeat the complicated in vitro experiments with protamines.

Figure 2 shows that in vitro spH2Bs compact free DNA similarly to somatic chromatin and figure 3 shows this chromatin has reduced nuclease accessibility. Nucleosomal chromatin cannot achieve the local and especially the long-range DNA density of protamine packaging [PMID 2043729]. We have improved the text to make this point more clearly and included several citations.

© 2023. Published by The Company of Biologists under the terms of the Creative Commons Attribution License (https://creativecommons.org/licenses/by/4.0/).

Major 3.

Fig. 2B is inadequate: samples should be run in the same gel or at least the internal gel ladder should be shown (not a manually drawn ladder). I also find the assay in Fig. 3C rather unconvincing, given that Tn5 tagmentation conditions can vary greatly, so two pictures seem skim evidence of reduced accessibility cause by spH2B histones (much less that they are the mechanisms directly responsible for this possible reduced accessibility). I must say, though, that the ApoI assay does seem to convincingly point at reduced accessibility.

Figures 3B and 3C have both been replaced to demonstrate what reviewers 2 and 3 have both pointed out. The panels now support the stated conclusions more robustly.

Major 4.

It would be interesting to quantify by RNA-seq the transcriptional effects cause by the spH2B mRNA / H2B.1 morpholino co-injection. E.g. to see if these effects are genome-wide or in particular loci. In addition, the authors could inject an spH2B mRNA with mutated SPKK motif residues to test the specificity of this aa motif in causing transcriptional silencing.

This is an interesting suggestion and we have been actively considering it as the next step in our investigation of sperm chromatin characteristics in Hydractinia. Nevertheless, we feel that the data presented support the conclusion of this paper about cell cycle arrest and a transcriptional block sufficiently.

Minor 5

Figure 1A is very confusing. Shouldn't "Histone-type" proteins should appear everywhere?

We have updated the title of figure 1 to make it clear that this figure addresses the evolution of sperm nuclear basic proteins (SNBPs) only.

We agree that animal genomes almost certainly all contain genes encoding multiple histone types. Coincidentally we have recently surveyed canonical histone gene types in all eukaryote genomes: https://www.biorxiv.org/content/10.1101/2021.05.12.443918v1

Minor 6

Are SPKK motifs found in other hydrozoan histones? (e.g. in H1 in sea urchin).

SPKK motif-containing histones are found in all hydrozoan transcriptomes that we have searched including Hydra and Clytia, but not in other cnidarians. This suggests that the motif has arisen in the Hydrozoan stem group, as represented in figure 1A.

Minor 7

Please make sure to include details on all the experimental methods (instead of referring to Török et al. 2016 publication).

We have gone to great lengths to ensure extensive methodological details are available in this manuscript for all parts of this work that are not already available. Equally full descriptions of the methods for the other experiments are in the cited article by the same first author as this work, and are available freely under Open Access. We are happy to reproduce them here if the editors feel this is appropriate.

© 2023. Published by The Company of Biologists under the terms of the Creative Commons Attribution License (https://creativecommons.org/licenses/by/4.0/).

Resubmission

First decision letter

MS ID#: DEVELOP/2022/201058

MS TITLE: Hydrozoan sperm-specific SPKK motif-containing histone H2B variants stabilise chromatin with limited compaction

AUTHORS: Anna Török, Martin JG Browne, Jordina C Vilar, Indu Patwal, Timothy Q DuBuc, Febrimarsa Febrimarsa, Erwan Atcheson, Uri Frank, Sebastian G Gornik, and Andrew Flaus

Thank you for resubmitting your above manuscript for consideration for publication to Development. Your manuscript has now been seen by two of the original Reviewers, who had assessed your paper before. I have now received their reports on the above manuscript, and have reached a decision.

The referees' comments are appended below, or you can access them online: please go to BenchPress and click on the 'Manuscripts with Decisions' queue in the Author Area.

As you will see, the referees continue to express considerable interest in your work, but some criticisms remain, which would be important to address before we can consider publication. If you are able to revise the manuscript along the lines suggested I will be happy receive a revised version of the manuscript.

In particular, and having looked through the manuscript myself, I share the view of the Reviewer 2, that an analysis of gene expression is important to substantiate the conclusions that transcription is affected. While I understand that the RNAseq requested by the Reviewer 2 may involve significant cost, I would understand if you are unable to perform RNAseq but at the very least suggest to perform RT-PCR for several genes known to be expressed by the embryo at these early stages of development.

In addition, the ATACsee dataset would need to be strenghtened, either by performing ATACseq, or alternatively, by providing additional quantifications and further representative images accordingly.

Lastly, both Reviewers request that the Methods section is rewritten with a more thorough explanation of the experimental details.

Your revised paper will be re-reviewed by one or more of the original referees, and acceptance of your manuscript will depend on your addressing satisfactorily the reviewers' major concerns. Please also note that Development will normally permit only one round of major revision. If it would be helpful, you are welcome to contact us to discuss your revision in greater detail. Please send us a point-by-point response indicating your plans for addressing the referee’s comments, and we will look over this and provide further guidance.

Please attend to all of the reviewers' comments and ensure that you clearly highlight all changes made in the revised manuscript. Please avoid using 'Tracked changes' in Word files as these are lost in PDF conversion. I should be grateful if you would also provide a point-by-point response detailing how you have dealt with the points raised by the reviewers in the 'Response to Reviewers' box. If you do not agree with any of their criticisms or suggestions please explain clearly why this is so.

Reviewer 1

Advance summary and potential significance to field

please see my first report

© 2023. Published by The Company of Biologists under the terms of the Creative Commons Attribution License (https://creativecommons.org/licenses/by/4.0/).

Comments for the author

The authors explain that they do not have access to the most appropriate, efficient and current methods of biophysical chromatin analysis and have therefore used some of the old techniques that were used in the 1980s/90s (despite the fact that even then there were more sophisticated methods).

This was quite surprising in the first version, but in the revised version the authors have made an effort to limit their conclusions and better explain their experimental approaches.

Given the interest of the study, I have no objection to its publication.

One minor point.

The authors need to better explain what somatic cells they used and how they isolated and manipulated them.

Reviewer 2

Advance summary and potential significance to field

Torok et al test the functional properties of SPKK-aminoacid motif containing H2B variands in hydrozoan cnidarians, more specifically: its role in reducing chromatin accessibility and in chromatin compactation. Unlike protamines or sea urchin SPKK H2B variants, the authors suggest that hydrozoan SPKK H2Bs restrict accessibility (and reduce transcription) without causing an overall compactation of the chromatin. The possibility that these different sperm-specific H2B variants, with distinct functional properties, have convergently evolved is of significance.

Comments for the author

I'm generally convinced by the author's response to my concerns/suggestions. This revised version involves mostly textual changes, but important modifications have been made in data presentation, particularly in Figure 3. On the downside, some important controls/comparisons (also suggested by other reviewers) like including protamine-compacted chromatin (maybe even sea urchin H2B-compacted sperm chromatin) in the same essays (Figure 3B), seem to be missins. This weakens the conclusions.

Also the results of the ATAC-see experiment are still rather unconvincing to me and I really think that including RNA-seq would greatly add in terms of understanding the transcriptional repression (is it global? or are specific genes/genomic particularly affected?).
Finally, I would encourage the authors to report in full detail the methods employed here.
Overall, though, I still find the manuscript an interesting contribution and, if additional experiments could be included, I would support its publication in Development.

First revision

Author response to reviewers' comments

Reviewer 1

The authors explain that they do not have access to the most appropriate, efficient and current methods of biophysical chromatin analysis and have therefore used some of the old techniques that were used in the 1980s/90s (despite the fact that even then there were more sophisticated methods). This was quite surprising in the first version, but in the revised version the authors have made an effort to limit their conclusions and better explain their experimental approaches. Given the interest of the study, I have no objection to its publication.

© 2023. Published by The Company of Biologists under the terms of the Creative Commons Attribution License (https://creativecommons.org/licenses/by/4.0/).

Reviewer 1 Comments for the Author

One minor point. The authors need to better explain what somatic cells they used and how they isolated and manipulated them.

Somatic cells in this context were obtained from pronase-dissociated feeding polyps. This includes all somatic cell types but not germ cells, since the latter are only present in sexual polyps but absent in feeding polyps [PMID 32054756]. This has been noted in the Methods.

Reviewer 2

Torok et al test the functional properties of SPKK-amino acid motif containing H2B variands in hydrozoan cnidarians, more specifically: its role in reducing chromatin accessibility and in chromatin compactation.

Unlike protamines or sea urchin SPKK H2B variants, the authors suggest that hydrozoan SPKK H2Bs restrict accessibility (and reduce transcription) without causing an overall compactation of the chromatin. The possibility that these different sperm-specific H2Bvariants, with distinct functional properties, have convergently evolved is of significance.

Reviewer 2 Comments for the Author:

I'm generally convinced by the author's response to my concerns/suggestions. This revised version involves mostly textual changes, but important modifications have been made in data presentation, particularly in Figure 3.

We thank the reviewer for recognising the effort that went into addressing all three of the previous reviewer comments in detail.

On the downside, some important controls/comparisons (also suggested by other reviewers) like including protamine-compacted chromatin (maybe even sea urchin H2B-compacted sperm chromatin) in the same essays (Figure 3B), seem to be missins. This weakens the conclusions.

In our response to a previous reviewer we explained that protamines are estimated to drive compaction of DNA in mouse sperm by 40 fold compared to somatic interphase nuclei [PMID 938720, 7297606] and produce chromatin that is 6 fold more compact than mitotic chromosomes [PMID 2043729], as recently reviewed by Okada [PMID 35344802]. In comparison, Figure 2 shows that in vitro spH2Bs compact free DNA similarly to somatic chromatin and figure 3 shows this chromatin has reduced nuclease accessibility. It is accepted that nucleosomal chromatin cannot achieve the local and especially the long- range DNA density of protamine packaging [PMID 2043729].

We agree with both this and the previous reviewer that a comparison with protamines could be interesting but we noted in a previous response that Hydractinia genomes do not encode detectable protamines or other sperm nuclear basic proteins (SNBPs), and that interpretation of native gel mobility assays could be difficult since protamines are likely to provide both more compaction and more charge neutralisation per unit length of DNA bound than histones. We feel that making a robust inter-species comparison between Hydractinia nucleosomal and sea urchin protamine compaction would require biophysical or imaging approaches that are beyond the scope of this manuscript.

Also the results of the ATAC-see experiment are still rather unconvincing to me and I really think that including RNA-seq would greatly add in terms of understanding the transcriptional repression (is it global? or are specific genes/genomic particularly affected?).

To provide a more convincing and quantitative demonstration of the ATAC-see experiment we have assembled a new supplementary figure 3 with 20 representative images of DNA (Hoechst stained) and ATAC-see for feeding polyp and sperm cells respectively captured under equivalent conditions. The majority of images for both feeding polyp (fig S3A) and sperm (fig S3B) cells contain punctate foci that we interpret as non-histone packaged mitochondrial DNA

© 2023. Published by The Company of Biologists under the terms of the Creative Commons Attribution License (https://creativecommons.org/licenses/by/4.0/).

effectively acting as a positive control. In contrast, the majority of feeding polyp but not sperm cells contain dispersed regions of signal in the ATAC-see channel. We then quantitated the ratio of total Hoechst and ATAC-see signal in each channel for each image and plotted this (fig S3C). This shows that the ATAC-see signal is much stronger relative to DNA for feeding polyps than sperm cells, and hence supports our interpretation that spH2Bs reduce DAN accessibility as also seen with our in vitro accessibility assays. We hope this provides a more convincing representation of the ATAC- see experimental results for the reviewer.

With respect to understanding the details of transcriptional repression, it is widely accepted that sperm are transcriptionally inactive [PMID 23856356] and that this global repression is mediated by chromatin [PMID 19759174]. In Figure 4J-N, we show by measuring EU incorporation that spH2Bs can arrest the cell cycle and block transcription as expected. We agree that the transcriptional block could be either the cause or consequence of cell cycle arrest. However, alternative corroborating measurements of global repression by RNA-seq or RT-PCR panel of RNA extracts would not readily address this question either. This is because of the lack of a housekeeping control gene that is confirmed to be unaffected by spH2Bs, so any quantitative transcriptomic approach would require an absolute quantitation to normalise RNA levels to input genomic DNA which in turn introduces significant technical challenges.

It is possible that differential packaging of distinct regions of the genome by spH2Bs results in local variation of transcription which could be identified by RNA-seq, although this is probably not tractable by a candidate-based RT-PCR panel given the many thousands of expressed genes at this stage. The microinjection and imaging for EU incorporation approach we developed simply demonstrates that cell cycle arrest and a major transcriptional block is caused by spH2Bs. We agree with the reviewer that identifying genes that are differentially repressed by spH2Bs would be interesting but this constitutes an entirely new study. The information would not contradict the data in Figure 4 and its interpretation in the text that spH2Bs, but not canonical H2B.1, can significantly reduce total transcription.

Finally, I would encourage the authors to report in full detail the methods employed here.

We appreciate that having full description of techniques in a single manuscript is useful for colleagues so we have inserted detailed descriptions of all the methods previously cited from our earlier publications. We have carefully reviewed the methods and inserted additional details about sources of materials.

Overall, though, I still find the manuscript an interesting contribution and, if additional experiments could be included, I would support its publication in Development.

We thank the reviewer and are delighted that both reviewers 1 and 2 agree that this work will be of interest to readers of Development.

Second decision letter

MS ID#: DEVELOP/2022/201058

MS TITLE: Hydrozoan sperm-specific SPKK motif-containing histone H2B variants stabilise chromatin with limited compaction

AUTHORS: Anna Török, Martin JG Browne, Jordina C Vilar, Indu Patwal, Timothy Q DuBuc, Febrimarsa Febrimarsa, Erwan Atcheson, Uri Frank, Sebastian G Gornik, and Andrew Flaus
ARTICLE TYPE: Research Report

I am happy to tell you that your manuscript has been accepted for publication in Development, pending our standard ethics checks.

© 2023. Published by The Company of Biologists under the terms of the Creative Commons Attribution License (https://creativecommons.org/licenses/by/4.0/).
